# Co-occurrence of *JAK2-V617 F* mutation and *BCR::ABL1* translocation in chronic myeloproliferative neoplasms: a potentially confounding genetic combination

**DOI:** 10.3389/fonc.2023.1329298

**Published:** 2024-01-12

**Authors:** Magda Zanelli, Alessandra Bisagni, Francesca Sanguedolce, Giuseppe Broggi, Valentina Fragliasso, Maurizio Zizzo, Andrea Palicelli, Giovanni Martino, Camilla Cresta, Cecilia Caprera, Matteo Corsi, Pietro Gentile, Fabrizio Gozzi, Domenico Trombetta, Paola Parente, Rosario Caltabiano, Nektarios Koufopoulos, Luca Cimino, Alberto Cavazza, Giulio Fraternali Orcioni, Stefano Ascani

**Affiliations:** ^1^ Pathology Unit, Azienda USL-IRCCS di Reggio Emilia, Reggio Emilia, Italy; ^2^ Pathology Unit, Policlinico Riuniti, University of Foggia, Foggia, Italy; ^3^ Department of Medical and Surgical Sciences and Advanced Technologies “G.F. Ingrassia” Anatomic Pathology, University of Catania, Catania, Italy; ^4^ Laboratory of Translational Research, Azienda USL-IRCCS di Reggio Emilia, Reggio Emila, Italy; ^5^ Surgical Oncology Unit, Azienda USL-IRCCS di Reggio Emilia, Reggio Emilia, Italy; ^6^ Pathology Unit, Azienda Ospedaliera Santa Maria di Terni, University of Perugia, Terni, Italy; ^7^ Hematology, Centro di Ricerca Emato-Oncologica (C.R.E.O.), University of Perugia, Perugia, Italy; ^8^ Ocular Immunology Unit, Azienda USL-IRCCS di Reggio Emilia, Reggio Emilia, Italy; ^9^ Laboratory Oncology, Fondazione IRCCS Casa Sollievo della Sofferenza San Giovanni Rotondo, San Giovanni Rotondo, Italy; ^10^ Second Department of Pathology, Medical School, National and Kapodistrian University of Athens, Attikon University Hospital, Athens, Greece; ^11^ Department of Surgery, Medicine, Dentistry and Morphological Sciences, University of Modena and Reggio Emilia, Modena, Italy; ^12^ Pathology Unit, Azienda Ospedaliera di Cuneo, Cuneo, Italy

**Keywords:** *BCR::ABL1*, *JAK2*, chronic myeloid leukemia, myeloproliferative neoplasm, polycythemia vera, primary myelofibrosis, essential thrombocythemia

## Abstract

Myeloproliferative neoplasms (MPNs) are classified into Philadelphia (Ph) chromosome–positive chronic myeloid leukemia (CML) and Ph-negative MPNs. *BCR::ABL1* translocation is the key genetic event of CML, whereas *JAK2/MPL/CALR* mutations are molecular aberrations of Ph-negative MPNs. Despite initially considered mutually exclusive genetic aberrations, the co-occurrence of *BCR::ABL1* and *JAK2* has been reported in a limited number of cases. The two genetic alterations may be identified either at the same time or *JAK2* aberration may be detected in patients with a previous CML treated with tyrosine kinase inhibitors or, finally, *BCR::ABL1* translocation occurs in patients with a history of *JAK2*-positive MPN. This combination of genomic alterations is potentially confounding with clinical manifestations often misinterpreted either as disease progression or drug resistance, therefore leading to inappropriate patient’s treatment. Our systematic review aims to improve hematologist and pathologist knowledge on this rare subset of patients. Starting from the presentation of two additional cases from our routine daily practice, we focus mainly on clinical, laboratory, and bone marrow histological findings, which may represent useful clues of *BCR::ABL1* and *JAK2* co-occurrence. The interaction between *JAK2* and *BCR::ABL1* clones during the disease course as well as therapy and outcome are presented.

## 1 Introduction

Myeloproliferative neoplasms (MPNs) are clonal disorders of hematopoietic stem cells with proliferation of one or more of the hematopoietic lineages, classified into distinct categories depending on different features, including the underlying genetic abnormalities (1–3). MPNs are classified into Philadelphia (Ph)–positive t(9;22) (q34.1;q11.2) chronic myeloid leukemia (CML) and Ph-negative MPNs. *BCR::ABL1* gene translocation is the key molecular event defining CML. The reciprocal rearrangement and fusion of the *BCR* gene on chromosome 22 and *ABL* gene on chromosome 9 leads to the production of an oncoprotein that can be p190, p210, or p230 depending on the breakpoint of *BCR::ABL*. The presence of *BCR::ABL1* translocation is strictly associated with CML, being an essential requisite for CML development and diagnosis. On the other hand, mutations in the Janus kinase 2 (*JAK2*) gene on exon 14, mostly at codon 617 (*JAK2 V617F*) or other activating *JAK2* mutations in exon 12 or, more rarely, mutations in the myeloproliferative leukemia (*MPL*) gene or in exon 9 of Calreticulin (*CALR*) gene are observed in Ph-negative MPNs, which may have different clinical and morphological features presenting as either polycythemia vera (PV), primary myelofibrosis (PMF), or essential thrombocythemia (ET). The gain-of-function *JAK2* mutation leads to the activation of the JAK/STAT signaling pathway, resulting in cellular proliferation and resistance to apoptosis. The *JAK2 V617F* mutation is detected in PV (95% of cases), PMF (60%), and ET (50%) (1–3). Although *BCR::ABL1* translocation and *JAK2* mutation were initially considered to be mutually exclusive, more recently the simultaneous presence of these genetic alterations has been reported (4–53). The co-occurrence of *JAK2 V617F* mutation and *BCR::ABL1* translocation in the same patient is a rare event with a frequency ranging from 0.2% to 2.5% according to different studies (21, 38, 54). The two genetic alterations may be identified either simultaneously or *JAK2* mutation may be found in the setting of a previously diagnosed CML treated with a tyrosine kinase inhibitor (TKI) or finally *BCR::ABL1* translocation may develop in patients with a long history of *JAK2*-positive MPN. Current scientific literature reports 85 cases of coexistence of *BCR::ABL1* and *JAK2* (4–53). Most reports are isolated cases or small case series and, given the paucity of data, clinical and pathological characteristics and prognosis of patients harboring concurrent *BCR::ABL1* and *JAK2* abnormalities have not been systematically evaluated. In the present report, starting from the presentation of two cases with a long history of *JAK2*-positive PMF subsequently developing CML with *BCR::ABL1* translocation acquisition, we performed a systematic review with the purpose to improve our understanding of this particular setting of MPNs with co-occurring *BCR::ABL1* translocation and *JAK2* mutation, focusing on clinical and laboratory signs and on bone marrow (BM) morphological features, which may represent clues of coexistence of Ph-negative MPNs and CML. Interaction between *JAK2* and *BCR::ABL1* clones during the disease course is also discussed as well as treatment and outcome.

## 2 Case descriptions

### 2.1 Case 1

**Figure 1:**
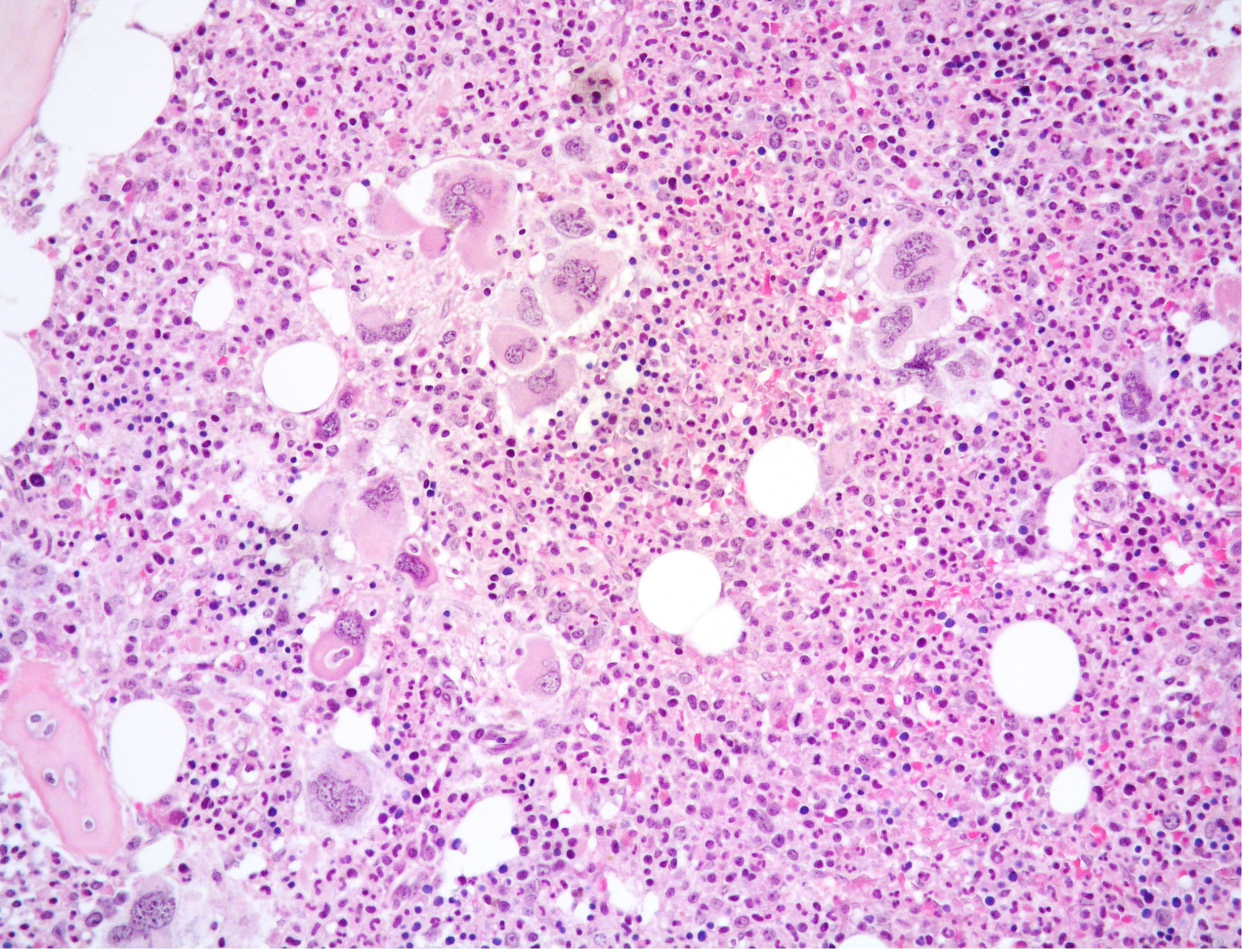
Medium power view of BM biopsy showing hypercellular marrow with normal-maturing prevalent granulopoiesis and clusters of variably sized MKs including large forms with bulbous nuclei (Hematoxylin and eosin, 200x magnification).

**Figure 2:**
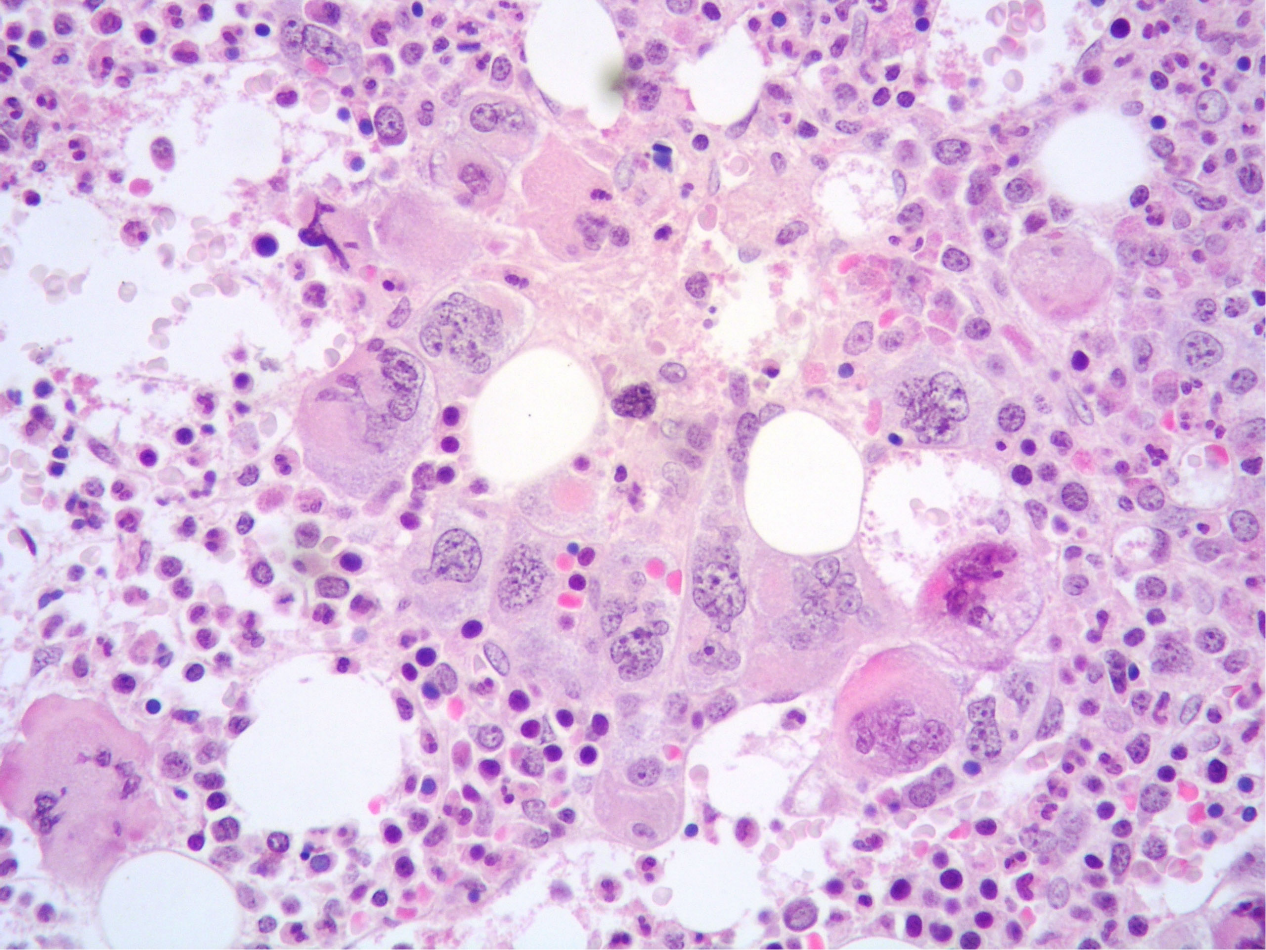
High power view of BM highlighting a dense cluster of variably-sized MKs (Hematoxylin and eosin, 400× magnification).

A 56-year-old woman presented to our center because of increasing white blood cell count (WBC 48.1 × 10^9^/L). The patient had a 13-year history of a Ph-negative, *JAK2*-positive MPN consistent with pre-fibrotic PMF. At the time of PMF diagnosis the patient had splenomegaly (spleen: 14 cm in diameter), with platelet (PTL) count up to 800 × 10^9^/L, whereas WBC and hemoglobin (Hb) level were within normal limits. Peripheral blood (PB) smear showed dacryocytes, poikilocytes, erythroblasts, and neutrophils with hyper-segmented nuclei. BM aspirate revealed giant megakaryocytes (MKs) and small MKs with hyperchromatic nuclei. BM biopsy showed an hypercellular marrow (80% cellularity) with reduced erythropoiesis and hyperplastic, normal-maturing granulopoiesis; variably sized MKs, including large elements with bulbous nuclei admixed with small-sized forms with hyperchromatic nuclei, were increased and showed loose and dense clusters (**Figures 1**, **2**). Reticulin fibrosis grade 0 was observed. Chromosomal analysis demonstrated a normal female karyotype (46XX) and molecular testing revealed V617F mutation in *JAK2* gene (allelic burden: 83.98%), whereas *CALR* and *MPL* mutations were absent as well as *BCR::ABL*1 translocation. Due to repeated cerebral thrombo-embolic episodes, the patient received antiaggregant therapy with acetylsalicylic acid (ASA) and hydroxyurea (HU), followed 8 years later by interferon (IFN) alpha with good control of the disease.

**Figure 3:**
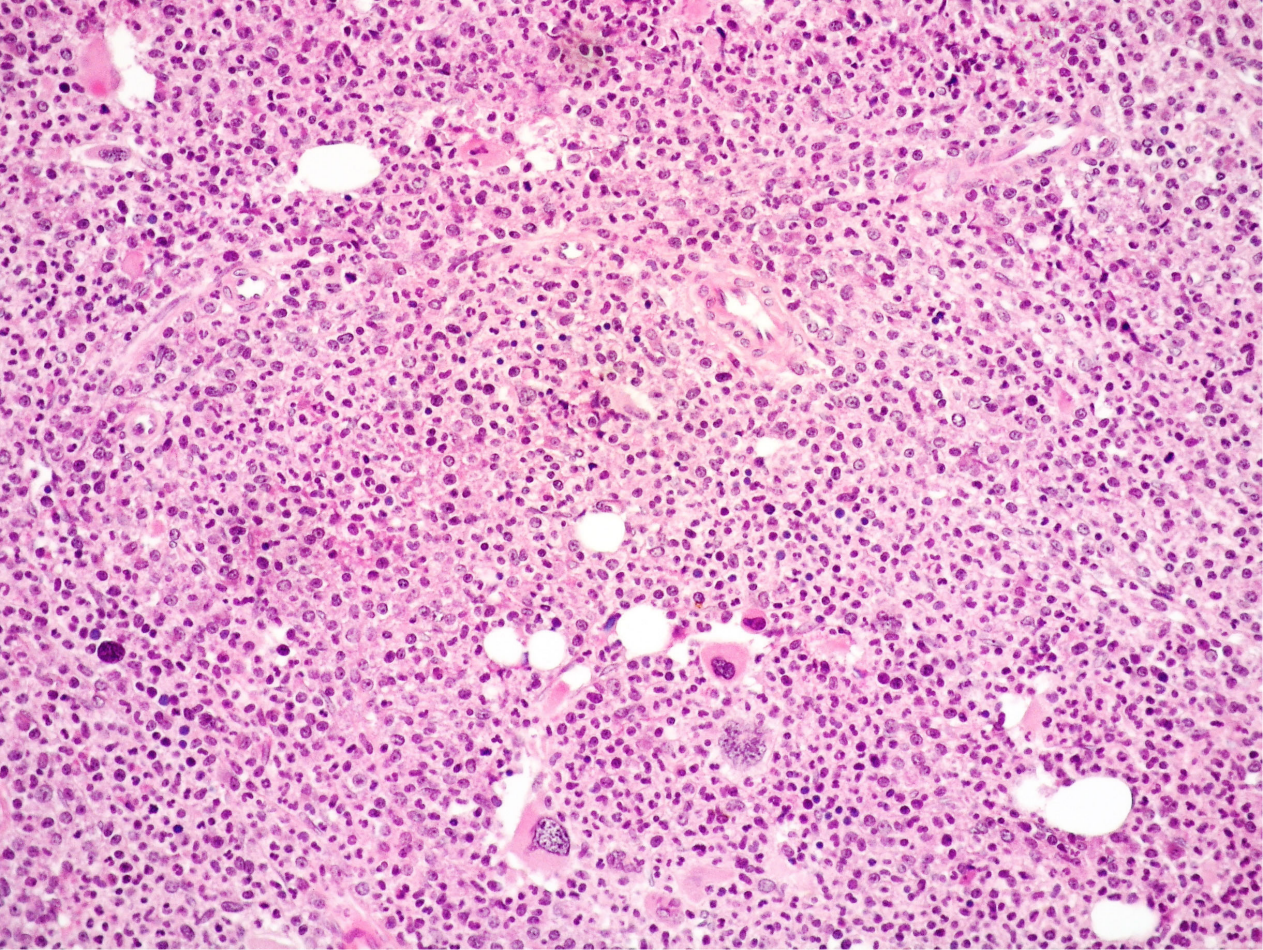
Medium power view of BM biopsy showing hypercellularity with high myeloid: erythroid ratio and mainly small MKs with scarce tendency to form clusters (Hematoxylin and eosin, 200× magnification).

At the time of referral to our center, blood tests showed progressively increasing leukocytosis up to 48 × 10^9^/L, with normal Hb level (13.3 g/dL) and normal PTL count (416 × 10^9^/L). Chromosomal analysis demonstrated Ph chromosome; fluorescent *in-situ* hybridization (FISH) analysis detected a *BCR::ABL1* fusion in 99% of interphase nuclei indicative of t(9:22)(q34;q11) translocation; quantitative reverse transcriptase–polymerase chain reaction (RT-PCR) for *BCR::ABL1* fusion transcripts demonstrated 177% *BCR::ABL1* fusion transcript of p210 variant. At this time, *JAK2 V617F* remained positive with an allelic burden of 21.48%; *MPL* and *CALR* remained negative. BM biopsy revealed hypercellularity with high myeloid: erythroid ratio and increased forms of intermediate maturation (metamyelocytes and band neutrophils); MKs were increased in number and predominantly small sized with scarce tendency to form loose clusters (**Figure 3**); no increase in CD34-positive hematopoietic precursors was noted. Reticulin fibrosis grade 0 was present. BM histopathology combined with clinical data and molecular results were suggestive of CML chronic phase (CP) with concomitant *JAK2* mutation and *BCR::ABL1* rearrangement. The patient was started on TKI Imatinib at 400 mg/die with a complete cytogenetic response (CCyR) and *BCR::ABL1* transcript lower than 10%, 3 months later. Despite a good response of the Ph-positive MPN, *JAK2 V617* allelic burden increased up to 89.83%. The marrow was hypercellular with granulocytic predominance and an increase in variably sized MKs with clustering. Grade 0 fibrosis was present. The pathological features were consistent with persistency of the JAK2-positive MPN. IFN-alpha (3 MU/every 10 days) was therefore re-introduced in association with Imatinib (400 mg/die) and ASA, obtaining a good control of the disease with deep molecular response (DMR) of CML, but *JAK2* still positive at a constant allelic burden (20%) at 7 years from CML diagnosis.

### 2.2 Case 2

An 82-year-old woman was referred to our center because of increasing leukocytosis (WBC 53.78 × 10^9^/L) with mild anemia (Hb 10.7 g/dL) and normal PTL count (399 × 10^9^/L). The patient had been diagnosed *JAK2 V617F*–positive (allelic burden: 27%) PMF 7 year earlier at another institution and had been treated with HU for few weeks only, due to drug intolerance (cutaneous edema). The patient had voluntarily interrupted hematologic follow-up for approximately 6 years before presenting to our center. At the time of initial PMF diagnosis, *BCR::ABL1* was negative. At the time the patient was referred to us, BM biopsy showed a markedly hypercellular marrow (95% cellularity) with significantly increased myeloid to erythroid ratio, normally maturing myeloid lineage and no increase in CD34-positive hematopoietic precursors. The erythroid lineage was markedly reduced as well as MKs, which were predominantly of small size. Grade 3 reticulin fibrosis was present. Quantitative RT-PCR identified *BCR::ABL1* p210 fusion gene (95%). Karyotyping showed 46XX and cytogenetic analysis revealed t(9;22)(q34;q11). FISH analysis identified *BCR::ABL1* fusion in 90% of interphase nuclei. *JAK2 V617F* mutation was confirmed (allelic burden: 30%). Splenomegaly (spleen: 14.5 cm in diameter) was identified. Therefore, the final diagnosis was CML in CP, arising in the background of a JAK2-positive MPN with grade 3 reticulin fibrosis consistent with fibrotic PMF. The patient was started on Imatinib at 100 mg/die, subsequently increased to 200 mg/die. Four months later, *BCR::ABL1* fusion gene declined to 18%. Despite a discrete CML control, due to increasing thrombocytosis (PTL count > 1.000 × 10^9^/L), a new BM biopsy was performed 5 months later. The marrow was hypercellular (80% cellularity) with a significant prevalence of the myeloid lineage and an increase in variably sized MKs even with hyperchromatic nuclei and a moderate tendency of dense clustering. Grade 3 reticulin fibrosis was present. *JAK2 V617F* mutation was confirmed with 15% allelic burden. The pathological features were in keeping with persistency of the JAK2-positive MPN with fibrosis. Molecular analysis showed persistence of *BCR::ABL1* fusion gene (15%). In addition to Imatinib (200 mg/die), the patient was put on recombinant IFN alpha-2b (750.000 U/die). Twelve months later, PTL count decreased to 500 × 10^9^/L with 5% *JAK2* allelic burden and a major molecular response (MR3) was achieved (*BCR-ABL1* fusion gene: 0.07%). The patient is still on treatment with both drugs with a good control of the disease at 24 months from CML diagnosis.

## 3 Methods of literature review

Our systematic literature review was carried out adhering to the Preferred Reported Items for Systematic Reviews and Meta-Analyses (PRISMA) guidelines. The search was conducted using PubMed/MEDLINE, EMBASE, Scopus, Cochrane Library (Cochrane Database of Systematic Reviews, Cochrane Central Register of Controlled Trials (CENTRAL), and Web of Science (Science and Social Science Citation Index) databases, with the following non-MeSH/MeSH terms “*JAK2*” AND *BCR::ABL1* coexistent” [Mesh]. The search was performed from 2005 when *JAK2* gene was first discovered to August 2023. The criteria for inclusion were as follows: (1) MPNs harboring concurrent *JAK2* mutation and *BCR::ABL1* translocation; (2) clinically relevant dual driver mutations representing coexistent Ph-positive CML and Ph-negative *JAK2*-positive MPN; (3) retrospective, observational case-control studies, case reports and/or series, literature review. The exclusion criteria were as follows: (1) studies not published in English; (2) lack of concurrent *JAK2* mutation and *BCR::ABL1* translocation (3) clinically non-relevant, incidentally detected *BCR-ABL1* translocation, in the context of a Ph-negative *JAK2*-positive MPN; (4) clinically non-relevant, incidentally detected *JAK2* mutation, in the context of a Ph-positive MPN. Two independent reviewers (M. Zanelli, AB) identified papers on the basis of title, abstract, and key words; then, they evaluated whether the selected papers met the inclusion criteria by reading the article full texts. Moreover, reference lists from each retrieved article were checked to find additional reports. From selected papers, the following information was collected: author’s surname, year of publication, patient’s age and sex, disease course, clinical and laboratory findings suggestive of a second coexistent MPN, BM histological features suggestive of a second coexistent MPN, interaction between *JAK2* and *BCR::ABL1* clones, treatment, and outcome. A third independent reviewer (AS) revised all collected results and solved discrepancies.

## 4 Results of literature data including our cases

### 4.1 Study selection and characteristics

**Figure 4:**
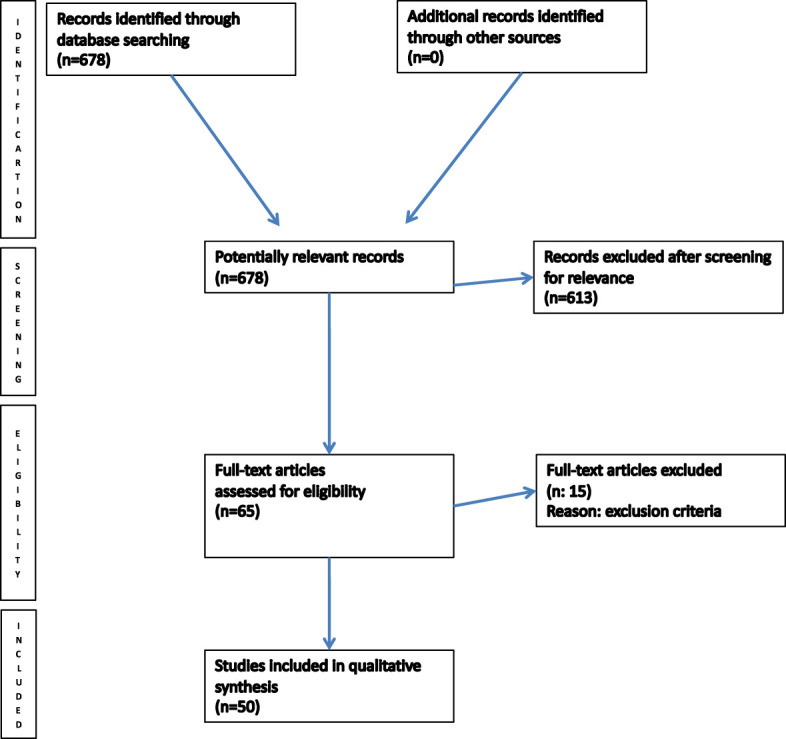
PRISMA flow chart of literature search.

The final literature search, performed in August 2023, identified 678 potential items of interest (**Figure 4**). After removing irrelevant publications (615), 65 records were further analyzed. Fifteen of these were excluded according to exclusion criteria, while 50 full-text articles were assessed for eligibility and included into qualitative synthesis. The included articles were case reports/case series (4–53).

### 4.2 Epidemiology

To date, 50 studies have analyzed MPNs with concomitant *JAK2* and *BCR::ABL1*, with a total of 85 cases (4–53). Including our two cases, a total of 87 cases have been described so far. Patient characteristics, course of the disease, clinical and laboratory data, as well as BM histological features suggestive of concomitant *JAK2* and *BCR::ABL1*, interaction between *JAK2* and *BCR::ABL1* clones, treatment and outcome, are summarized in **Supplementary Table S1**.

Patients with coexistent *JAK2* and *BCR::ABL1* were aged between 24 and 87 years with an average of 60.1 years. Males were slightly more frequently affected than females (46/87; 52.87%). Including our cases, 28 patients had PV, 24 PMF, 20 ET, 10 MPNs not otherwise specified (MPN, NOS), three post-polycythemia vera myelofibrosis (PPV-MF), one post-essential thrombocythemia myelofibrosis (PET-MF) and in one case the initial diagnosis was unavailable.

**Figure 5:**
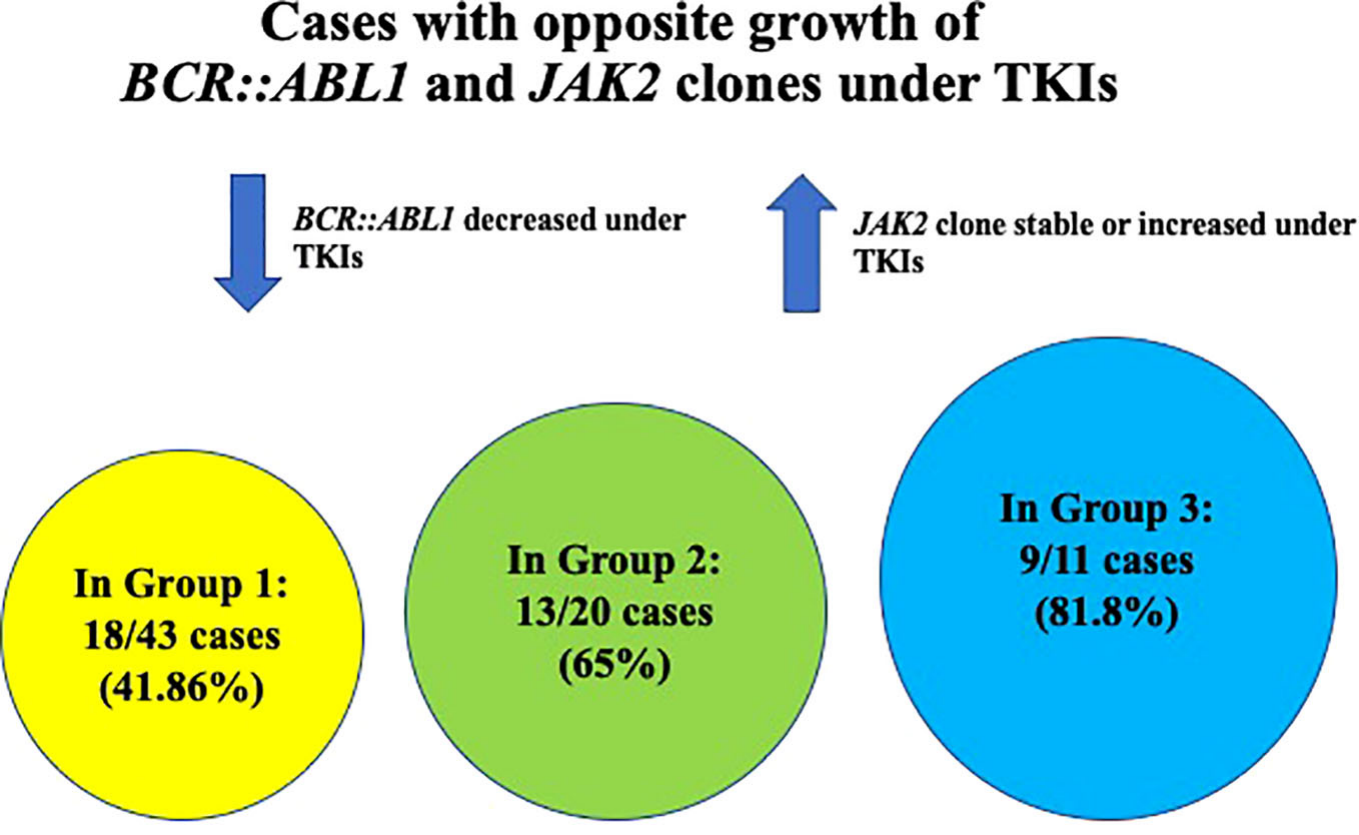
Graphic showing the frequency of cases with opposite growth of *BCR::ABL1* and *JAK2* clones under TKI treatment in the three different groups of patients.

Three different settings of patients were observed: Group 1: Ph-negative MPN preceded CML in 43 cases (43/87; 49.42%) (6, 8, 13, 15–17, 21–23, 27, 28, 31, 32, 35, 37–39, 41–43, 45–51), Group 2: CML preceded Ph-negative MPN in 20 cases (20/87; 22.98%) (4, 5, 9, 10, 15, 18, 19, 22, 24–26, 38, 39, 46, 48) and Group 3: CML and Ph-negative MPN were diagnosed concomitantly in 24 cases (24/87; 27.58%) (7, 10–12, 14, 15, 20, 22, 23, 29, 30, 33, 34, 36, 37, 39, 40, 44). Data on the distribution of Ph-negative MPNs in the three groups of patients are summarized in **Table 1**. Data on different types of TKIs used in this setting of patients and data on the limited number of cases receiving allogenic stem-cell transplantation (allo-SCT) are summarized in **Tables 2** and **3**, respectively. Data on the frequency of cases showing an opposite growth between *BCR::ABL1* and *JAK2* clones under TKI treatment in the three groups are showed in **Figure 5**.

**Table 1:** Distribution of Ph-negative MPNs in the three groups of patients with concomitant or sequential JAK2-positive MPN and CML (see **Supplementary Material**).

Frequency of different groups of Ph-negative MPN+ CML patients	Frequency of PV	Frequency of ET	Frequency of PMF	Frequency of PPV-MF	Frequency of PET-MF	Frequency of MPN,NOS	Frequency of MPN with NA diagnosis
**Group 1:** **Ph-negative MPN,** **then CML** 43/87 (49.42%)	20/43 (46.51%)	12/43 (27.90%)	8/43 (18.60%)	1/43 (2.32%)	1/43 (2.32%)		1/43 (2.32%)
**Group 2:** **CML, then Ph-negative MPN** 20/87 (22.98%)	5/20 (25%)	2/20 (10%)	6/20 (30%)			7/20 (35%)	
**Group 3: concomitant CML and Ph-negative MPN** 24/87 (27.58%)	3/24 (12.5%)	6/24 (25%)	10/24 (41.66%)	2 (8.3%)		3/24 (12.5%)	

CML, chronic myeloid leukemia; ET, essential thrombocythemia; MPN, myeloproliferative neoplasm; MPN, NOS, myeloproliferative neoplasm not otherwise specified; NA, not available; PET-MF, post-essential thrombocythemia myelofibrosis; PMF, primary myelofibrosis; PPV-MF, post-polycythemia vera myelofibrosis; PV, polycythemia vera.

**Table 2:** Data on different TKIs used in concomitant or sequential JAK2-positive MPNs and CML (see **Supplementary Material**).

Single or multiple lines of TKIs	N° of patients receiving different TKI lines/total cases treated with TKIs (Ref)	N° of patients with DMR (Ref)	N° of patients with CCyR (Ref)	N° of patients with hematological Response (Ref)	N° of patients with NA data on TKI response (Ref)	N° of patients with poor response to therapy (Ref)
**Imatinib**	43/81 patients (53.08%) (Ref 4–9, 11–15, 17–20, 22, 25, 27, 34–41, 46, 50, 53 present report case 1and case 2)	19/43 patients (44.18%) (Ref 4, 8, 9, 11, 15, 17, 20, 25, 27, 34, 38, 40, 41, 46, 51, 53)	5/43 patients (11.62%) (Ref 5, 18–20, present report case 1and case 2)	2/43 patients (4.65%) (Ref 12, 39)	12/43 patients (27.90%) (Ref 6, 7, 13, 14, 22, 33, 35–37)	5/43 patients (11.62%) (Ref 38, 39, 50)
**Dasatinib**	3/81 patients (3.70%) (Ref 31, 44, 47)	1/3 patient (33.33%) (Ref 44)	1/3 patient (33.33%) (Ref 31)	1/3 patient (33.33%) (Ref 47)		
**Nilotinib**	7/81 patients (8.64%) (Ref 38, 39, 46, 48, 48, 51)	4/7 patients (57.14%) (Ref 39, 46, 48, 51)				3/7 patients (42.85%) (Ref 38, 48)
**Imatinib, Dasatinib**	10/81 patients (12.34%) (Ref 10, 21, 22, 24, 26, 37, 39, 43)	5/10 patients (50%) (Ref 10, 24, 26, 39, 43)		1/10 patient (10%) (Ref 21)	2/10 patients (20%) (Ref 22, 37)	2/10 patients (20%) (Ref 3, 39)
**Imatinib, Nilotinib**	3/81 patients (3.70%) (Ref 10, 25, 39)	1/3 patient (33.33%) (Ref 25)	1/3 patient (33.33%) (Ref 10)			1/3 patient (33.33%) (Ref 39)
**Nilotinib, Dasatinib**	1/81 patient (1.23%) (Ref 28)	1/1 patients (100%) (Ref 28)				
**Imatinib, Ponatinib**	2/81 patients (2.46%) (Ref 38, 48)					2/2 patients (100%) (Ref 38, 48)
**Imatinib, Bosutinib**	2/81 patients (2.46%) (Ref 38, 39)	1/2 patient (50%) (Ref 38)				1/2 patient (50%) (Ref 39)
**Imatinib,** **Fumatinib**	1/81 patient (1.23%) (Ref 49)				1 patient (100%) (Ref 49)	
**Imatinib, Dasatinib, Nilotinib**	4/81 patients (4.93%) (Ref 16, 22, 39, 42)				3/4 patients (75%) (Ref 16, 22, 42)	1/4 patient (25%) (Ref 39)
**Imatinib, Nilotinib, Ponatinib**	1/81 patient (1.23%) (Ref 32)					1 patient (10%) (Ref 32)
**Imatinib, Nilotinib, Sunitinib, Ponatinib,** **Dasatinib**	1/81 patient (1.23%) (Ref 48)	1 patient (100%) (Ref 48)				
**Imatinib, Dasatinib, Nilotinib, Bosutinib, Ponatinib**	1/81 patient (1.23%) (Ref 52)	1 patient (100%) (Ref 52)				
**Unspecified TKI**	2/81 patients (2.46%) (Ref 29, 37)				2 patients (100%) (Ref 29, 37)	

CCyR, complete cytogenetic response; CML, chronic myeloid leukemia; DMR, deep molecular response; MPN, myeloproliferative neoplasm; NA, not available; Ref, reference; TKI, tyrosine kinase inhibitor.

**Table 3:** Allo-SCT in concomitant or sequential JAK2-positive MPNs and CML (see **Supplementary Material**).

	Number of cases (Reference)	Type of MPN	Other treatment (s) administered	Outcome
**Group 1:** **Ph-negative MPN, then CML**	1 (Ref 45) 1 (Ref 46) 1 (Ref 48) 1 (Ref 48)	PV ET, then PET-MF PMF PMF	Phlebotomy, Hu, Ruxolitinib, spleen RT IFN, phlebotomy, anagrelide, imatinib, HU HU, Imatinib, Nilotinib, Sunitinib, Ponatinib, Dasatinib, Omacetaxine, Ruxolitinib, splenectomy Ruxolitinib, Nilotinib	DMR 4 months after allo-SCT DMR 24 months after allo-SCT DMR 49 months after allo-SCT DMR 17 months after allo-SCT
**Group 2** **CML, then Ph-negative MPN**	1 (Ref 46)	PMF	Nilotinib, ruxolitinib	DMR at 4 months after allo-SCT
**Group 3** **Concomitant CML and Ph-negative MPN**	1 (Ref 39) 1 (Ref 44)	PMF PMF	Imatinib, Dasatinib IFN, HU, Idarubicin cytarabine, Dasatinib	DOD DMR, outcome NA

Allo-SCT, allogenic stem cell transplantation; CML, chronic myeloid leukemia; DMR, deep molecular response; DOD, died of disease; ET, essential thrombocythemia; HU, hydroxyurea; INF, interferon; MPN, myeloproliferative neoplasms; NA, not available; PET-MF, post essential thrombocythemia myelofibrosis; Ph-negative, Philadelphia negative; PMF, primary myelofibrosis; PV, polycythemia vera; RT, radiotherapy.

### 4.3 Group 1 (Ph-negative MPN preceding CML): clinical symptoms and laboratory signs at second disease (CML) occurrence

In Group 1 (43 cases of Ph-negative MPN preceding CML), the Ph-negative MPNs were distributed as follows: 20 PV, 12 ET, 8 PMF, 1 PPV-MF, 1 PET-MF; one case with unavailable MPN diagnosis.

At CML occurrence, WBC increase was the most common laboratory sign, observed in 38 of 42 cases with available data (4–43, 45–51), followed by anemia in 17/42 (6, 16, 17, 23, 27, 31, 37–39, 45, 50); basophilia in 17–42 (6, 13, 17, 23, 27, 35, 38, 41, 45, 47–50); PTL decrease in 9/42 (13, 27, 37–39, 45); leukoerythroblastosis in 7/42 (17, 31, 43, 45, 48); PTL increase in 6/42 (6, 22, 23, 37, 39, 48); eosinophilia in 6/42 (13, 41, 45, 47, 48), and LDH increase in 2/42 (27, 48). In this group, splenomegaly was detected in just under half of cases (20/42) (6, 8, 13, 16, 17, 23, 27, 28, 31, 32, 43, 45, 48–51) and hepatomegaly in 4/42 (6, 27, 49); systemic symptoms were observed only in a minority of patients as follows: fatigue/feeling unwell in 7/42 (6, 16, 31, 48, 51); weight loss (31, 48) and shortness of breath in 2/42 (6); dyspnea on exertion in 1/42 (27) and decreased appetite in 1/42 (48).

### 4.4 Group 1: histological features of BM biopsy performed at second disease (CML) occurrence

In Group 1, BM histology performed at the time of CML occurrence in 35/43 cases showed the following features: CML, CP in the majority of cases (13/35) (15–17, 21, 27, 32, 38, 39, 42, 43, 51); CML accelerated phase (AP) in 3/35 (6, 39, 50); CML+fibrosis in 7/35 (31, 41, 47–49), hence a total of 23/35 cases showed histological features reminiscent of CML; PET-MF histology in 3/35 (22, 23, 46); PPV-MF histology in 2/35 (48, 51); PMF histology in 1/35 (28); whereas hybrid features were observed in the remaining cases as follows: PPV-MF+CML in 3/35 (39, 45) with hybrid MKs both hypo-lobate and large hyper-lobate detected in 1/3 (45); PET-MF+CML in 2/35 (38, 39) with hybrid MKs in 1/2 (38); CML with hybrid MKs in 1/35 cases (48).

Of note, our case n°1 with CML (CP) histology was re-biopsied after having achieved a CCyR of CML with TKI; at this time, *JAK2* allele burden increased and histology switched to a PMF phenotype. Our case n°2 was re-biopsied at the time of CML partial MR and the histology switched from CML+fibrosis to a phenotype suggestive of JAK2+MPN with fibrosis, in particular the MKs from small-sized, non-clustering forms changed to variably sized, clustering MKs.

### 4.5 Group 1: *BCR::ABL1* and *JAK2* clone interaction

In Group 1, the following interaction between *BCR::ABL1* and *JAK2* clones were observed at the time of CML occurrence and during the course of the disease: the majority of cases (18/43; 41.86%) showed an opposite growth of the two genomic alterations with *BCR::ABL1* decrease under TKI and *JAK2* increase. In particular, 13/18 cases were positive for both *BCR::ABL1* and *JAK2* at CML occurrence and, under TKI, *BCR::ABL1* decreased (resulting often in CMR of CML) and *JAK2* increased (8, 13, 15, 21, 28, 31, 38, 39, 41, 49, 51); additionally, in 2/13 cases, *JAK2* increase was clinically associated with concomitant hematocrit (HCT) increase (8) or with concomitant PTL and WBC increase (31); in 4/18 cases, *BCR::ABL1* was positive and *JAK2* negative at CML occurrence and, under TKI, an opposite growth of the two genomic alteration was seen, with *BCR::ABL1* decrease and *JAK2* increase (16, 17, 27, 43); in all these four cases, *JAK2* increase was associated with clinical PV recurrence; in 1/18, at CML occurrence *BCR::ABL1* was positive and *JAK2* not evaluated and under TKI, an opposite growth of *BCR::ABL1* and *JAK2* was observed similarly to the above mentioned cases (46). In 3/43 cases, *BCR::ABL1* and *JAK2* were both positive at CML occurrence and presented a similar growth either remaining both positive and at the same allele burden before and after TKI (1/3 cases) (39), or both increasing during the disease course, without TKI (1/3 cases) (45) or both decreasing under TKI (1/3 cases). Finally, in 21/43 cases the interaction between *BCR::ABL1* and *JAK2* during the course of the disease was unavailable (6, 22, 23, 32, 35, 37–39, 42, 48, 50, 51).

### 4.6 Group 1: treatment and outcome

In this paragraph, as well as in the following paragraphs on treatment for Groups 2 and 3, the type of Ph-negative MPN preceding CML is indicated in brackets for each single case.

TKI therapy was administered in 41/43 cases; of 2/43 not receiving TKI, ASA, HU, and thalidomide were administered in 1/2 (ET) with unavailable outcome (23), whereas phlebotomy, HU, ruxolitinib, spleen radiotherapy, and allo-SCT in 1/2 (PV), with undetectable genomic markers at 4 months after SCT (45). TKI alone was administered in 1/41 cases (PMF) with DMR of CML (28). In the remaining cases, TKI was combined with other treatments as follows: TKI and phlebotomy in 3/41 cases with CCyR of CML in 1/3 (PV) and DMR of CML in 1/3 (PV) with no further data on outcome (8, 51), whereas in 1/3 cases (PV), outcome was unavailable (16); TKI and HU in 3/41 cases, with death of disease (DOD) in 1/3 (PV) (39) and unavailable outcome in 2/3 cases (1 PMF and 1 ET) (13, 37); TKI and IFN in 1/41 cases (PV) with death for AML 34 months after CML (15); TKI, HU and IFN in 3/41 cases with DMR of CML and good control of disease at 24 months from CML in 1/3 cases (PMF, present report case 2), good control of blood parameters in 1/3 (ET), which is under evaluation for SCT (22) and outcome unavailable in 1/3 (PET-MF) (37); TKI, HU and phlebotomy in 2/41 with DMR of CML and good control of PV in both cases (17, 27); TKI, HU and ASA in 1/41 (ET) with complete hematologic remission (47); TKI, HU, ASA, and phlebotomy in 2/41 (PV) with DMR of CML (41, 43); TKI, HU, and pipobroman in 1/41 cases (PV) with only hematologic response (21); TKI, HU, IFN, and ASA in 1/41 (PMF) with good control of diseases at 7 years from CML (PMF, present report case 1); TKI and ruxolitinib in 2/41, with DMR of CML in 1/2 (ET and PET-MF) and unavailable outcome in 1/2 (PPV-MF) (37, 38); TKI, ruxolitinib and phlebotomy in 1/41 (PV), with unavailable outcome (49); TKI, IFN and ruxolitinib in 1/41 (PV and PPV-MF) with suboptimal response and unavailable outcome (38); TKI, HU and ruxolitinib in 3/41 with DMR of CML in 1/3, but patient alive with disease (PMF and PET-MF) in 2/3 cases (39) and scarce response to therapy in 1/3 (PMF) (50); TKI, HU, ruxolitinib and phlebotomy in 1/41 (PV), with death for sepsis (48); TKI, HU, IFN, ruxolitinib in 1/41 (ET) with patient under evaluation for radiotherapy and SCT (49); TKI, HU, IFN, ruxolitinib, and phlebotomy in 1/41 (PV) with patient alive with disease (39); TKI and anagrelide in 1/41 (initial diagnosis unavailable) with remission (39); TKI, HU, and anagrelide in 1/41 (ET) with no response to therapy (48); TKI, phlebotomy and anagrelide in 1/41 (PV) with unavailable outcome (6); TKI, HU, anagrelide, IFN and ASA in 1/41 (ET) with unavailable outcome (42); TKI, HU, anagrelide, ruxolitinib, and ASA in 1/41 (PV) with CCyR of CML (31); TKI, HU, anagrelide, radioactive phosphorus in 1/41 (PV), with death for cerebral event 4 months after CML diagnosis (6); TKI, anagrelide, ruxolitinib and cytarabine in 1/41 (ET) with evolution to CML (BP) and death (38); TKI, HU, anagrelide, cytarabine and ruxolitinib in 1/41 (ET) with evolution to CML (BP) and poor response to therapy (32); TKI, HU, thalidomide and ruxolitinib in 1/41 (PV) with death for disease (39); TKI, phlebotomy, pipobroman, HU, spleen radiotherapy, IFN and ruxolitinib in 1/41 (PV) with death for gastric cancer (35); TKI, ruxolitinib and allo-SCT in 1/41 (PMF) with no detectable genomic markers 17 months after CML (48); TKI, omacetaxine, HU, ruxolitinib, splenectomy and allo-SCT in 1/41 (PMF) with no detectable genomic markers 49 months after SCT (48); TKI, IFN, anagrelide, HU, phlebotomy and allo-SCT in 1/41 (ET) with complete remission of both diseases at 24 months after allo-SCT (46).

### 4.7 Group 2 (CML preceding Ph-negative MPN): clinical symptoms and laboratory signs at second disease (Ph-negative MPN) occurrence

In Group 2 (20 cases), the Ph-negative MPNs occurring as second disease after CML, were distributed as follows: seven cases were JAK2+MPN, NOS; six cases PMF; five cases PV and two cases ET.

Laboratory signs detected in six cases of JAK2+MPN, NOS with available data occurring after CML were as follows: PTL increase in 4/6 cases (26, 38, 39); WBC increase in 3/6 (19, 22) and LDH increase in 1/6 (26). Of note in in 2/4 cases with PTL increase and in 1/3 cases with WBC increase, these laboratory signs were present despite good MR of CML (22, 26, 38).

Laboratory signs observed in five cases of PMF with available data occurring after CML were as follows: WBC increase in 3/5 (4, 15); PTL increase in 2/5 (10, 46); PTL decrease in 2/5 (4, 48); LDH increase in 2/5 (4, 15); anemia and immature myeloid cells in PB in 1/5 (48). In this group, splenomegaly was detected in 3/5 cases (4, 15, 48). Of note, in 1/2 cases with PTL increase, this laboratory sign was present despite good MR of CML (46).

Laboratory signs detected in the 5 cases of PV occurring after CML were as follows: red blood cell (RBC) increase and HCT increase in 2/5 cases (5, 24); WBC, Hb level and PTL increase in 1/5 cases (18); HCT and Hb increase in 1/5 (53), whereas HCT and Hb increase were associated with low erythropoietin (EPO) level in 1/5 (52).

Laboratory signs detected in the two cases of ET occurring after CML were as follows: PTL increase in 2/2 cases despite CCyR of CML (25); WBC increase in 1/2 despite CCyR of CML (25).

### 4.8 Group 2: histological features of BM biopsy performed at second disease (Ph-negative MPN) occurrence

BM biopsy performed at the time of Ph-negative MPN occurrence showed different features according to the type of Ph-negative MPN.

BM biopsy performed in six cases of PMF occurring after CML, showed the typical PMF histology (4, 9, 10, 15, 46, 48). Of note, in 2/6 cases, PMF histological features became clearly evident after CMR of CML with TKI; at the time PMF histology was detected, *BCR::ABL1* was negative and *JAK2* positive, at a constant allele burden in one case (4) and at an increased allele burden in the other case (9). Moreover, in 1/2 cases with PMF histology obvious after TKI, the presence of fibrosis at initial CML diagnosis could have been a possible clue for PMF, although fibrosis alone is not sufficient for PMF diagnosis as fibrosis may be found even in CML; in this case, *BCR::ABL1* and *JAK2* were both present from initial CML diagnosis, however PMF features became obvious after CMR of CML (4).

BM biopsy in five cases of JAK2-positive MPN, NOS occurring after CML with available histology, showed histological features consistent with Ph-negative MPN in all cases, in particular increase of large, clustering MKs was detected (15, 22, 38, 39). Of note, in 2/5 cases Ph-negative phenotype became evident after TKI, with *BCR::ABL1* decrease and *JAK2* increase (15, 22); in addition, in one of these two cases, BM performed at the time of initial CML diagnosis retrospectively revised showed not only MKs with hypo-lobate nuclei, typical of CML, but even large and occasionally clustering MKs with bulbous nuclei (22).

BM biopsy, available in 1/5 cases of PV occurring after CML, showed typical PV histological features (18). BM biopsy, available 1/2 cases of ET occurring after CML, showed MK increase (25).

### 4.9 Group 2: *BCR::ABL1* and *JAK2* clone interaction

In Group 2, the majority of cases (13/20; 65%) showed an opposite growth of *BCR::ABL1* and *JAK2* as follows: in 4/20 cases, *BCR::ABL1* and *JAK2* were coexistent at the time of CML and under TKI, *BCR::ABL1* decreased whether *JAK2* increased (4, 5, 9, 26); in 11/20 cases, only *BCR::ABL1* was present at CML diagnosis and *JAK2* was either negative (6/11 cases) (18, 25, 48, 52, 53) or not evaluated (5/11 cases) (19, 22, 38, 46); under TKI, *BCR::ABL1* decreased with CMR and *JAK2* progressively increased. In 3/20, both *BCR::ABL1* and *JAK2* decreased under TKI showing a similar growth (10, 15, 24) and in 2/20 cases clone interaction was unavailable (15, 39).

### 4.10 Group 2: treatment and outcome

TKI treatment was administered in 19/20 cases and of these cases, TKI alone was administered in 2/19 cases (PMF), both of which achieved DMR of CML (9, 10). TKI and HU were administered in 3/19 cases, of which, 1/3 (MPN, NOS) achieved CCyR of CML (26), 1/3 (MPN, NOS) achieved CMR of CML and good control of PTL count (38) and 1/3 (MPN, NOS) died of disease (CML-BP), one year after diagnosis (38). TKI and IFN were administered in 2/19 cases, of which one (MPN, NOS) achieved CCyR of CML (19) and one (MPN, NOS) was alive with disease (39); TKI and hydroxycarbamide in 1/19 (PMF) with DMR of CML (4); TKI, IFN, HU and phlebotomy in 2/19, of which one (PV) achieved CCyR of CML and one (PV) achieved negativity of both genomic markers (5, 24); TKI, IFN, HU, phlebotomy and ASA in 2/19 (PV) with DMR of CML and good control of disease (52, 53); TKI, anagrelide and HU in 1/19 (MPN, NOS) with DMR of CML (15); TKI, IFN, HU and cytosine arabinoside in 1/19 (PV) with DMR of CML (18); TKI, anagrelide, HU and IFN in 1/19 (ET) with CCyR of CML, good hematological parameters, but *JAK2* constantly positive (25); TKI, HU and ruxolitinib in 1/19 (PMF) with scarce response to therapy (48); TKI and anagrelide in 1/19 (ET) with CCyR of CML and good clinical conditions, but *JAK2* constantly positive at 3 years from CML diagnosis (25); TKI, ruxolitinib and allo-SCT in 1/19 (PMF), with complete remission at 4 months after allo-SCT (46); TKI, HU and spleen radiotherapy in 1/19 (MPN, NOS), with death for pneumonia (22). In 1/20 cases (PMF), HU and IFN were administered without TKI and outcome is unavailable (15).

### 4.11 Group 3 (coexistent CML and Ph-negative MPN): clinical and laboratory signs

Of Group 3 (24 cases of concomitant CML and Ph-negative MPN), 10 cases were coexistent PMF+CML; six cases coexistent ET+CML; three cases coexistent MPN, NOS+CML, three cases coexistent PV+CML, and two cases coexistent PPV-MF+CML.

Clinical symptoms and laboratory signs observed in eight cases of coexistent PMF+CML with available data were as follows: WBC increase in 6/8 (12, 33, 39, 40, 44); anemia in 5/8 (7, 14, 39, 44); PTL increase in 5/8 (12, 14, 20, 40, 44); splenomegaly in 5/8 (7, 14, 20, 40, 44); basophilia in 2/8 (12, 33), high LDH in 2/8 (33 ,44), weight loss 1/8 (40), night sweats in 1/8 (40), pruritus in 1/8 (40), hepatomegaly in 1/8 (40) and leukoerythroblastosis in 1/8 (33).

Laboratory signs observed in six cases of coexistent ET+CML were as follows: WBC increase in 4/6 cases (23, 30, 36, 39); PTL increase in 5/6 (20, 23, 30, 34); basophilia in 2/6 (23, 36) and anemia in 1/6 (23).

Laboratory signs detected in three cases of coexistent MPN, NOS+CML were as follows: WBC increase in 1/3 cases (15); anemia in 1/3 (29), and, finally, high PTL despite TKI in 1/3 cases (36).

Laboratory signs and clinical symptoms observed in three cases of coexistent PV+CML were as follows: high WBC in 3/3 cases (11, 22, 39); high HB level in 1/3 (11), high HCT in 1/3 (11), EPO level below normal level in 1/3 (11), basophilia and eosinophilia in 1/3 cases (22) as well as splenomegaly (22). Of note, after TKI, despite WBC decrease, Hb level and HCT were found to be increased in 1/3 cases (22).

Laboratory signs detected in two cases of coexistent PPV-MF+CML were as follows: splenomegaly in 2/2 cases (37); anemia in 1/2 (37) and WBC increase in 1/2 (37).

### 4.12 Group 3: histological features of BM biopsy

In Group 3, BM histology showed the following features subdivided according to the type of Ph-negative MPN associated with CML.

BM biopsy of the 10 cases of coexisting CML+PMF showed the following features: a mixed histology CML+PMF in 4/10 cases (33, 39, 40); BM fibrosis in 2/10 cases (7, 10); BM fibrosis+MK hyperplasia in 2/10 (12, 20); PMF histology in 1/10 (44); CML + fibrosis in 1/10 (14). Of note, 1/3 cases with mixed histology CML+PMF and the case with CML+fibrosis were re-biopsied after TKI and showed a switch to PMF histology (14).

BM biopsy was available in 5/6 cases with coexistent CML+ET. A mixed histology of CML+ET was present in 2/5 cases (36, 39), CML (CP) in 1/5 (20) and MK hyperplasia in 1/5 (23), whereas in 1/5, a CML histology was observed pre-TKI therapy, with a shift to ET-histology post-TKI therapy (34).

BM biopsy was available in 2/3 cases with coexistent CML+PV; a mixed histology of CML+PV was present in 1/2 (39) and MF histology in 1/2 (22).

BM biopsy of the three cases of coexistent CML+JAK2-positive MPN, NOS showed a mixed histology combining CML features+ clustering large MKs in 2/3 (15, 29) and panmyelosis with MK clustering in 1/3 (36).

BM biopsy data were incomplete in two cases of coexistent CML+PPV-MF and only grades 1 and 2 fibrosis was reported (37).

### 4.13 Group 3: *BCR::ABL1* and *JAK2* clone interaction

In group 3, *BCR::ABL1* and *JAK2* were detected at diagnosis, but clone interaction during the course of the disease was unavailable in 13/24 cases (14, 29, 30, 34, 36, 37, 39, 44). In 9/11 cases (81.8%) with available data on clone interaction, *BCR::ABL1* and *JAK2* showed an opposite growth as, under TKI, *BCR::ABL1* decreased and *JAK2* either remained constant or increased (7, 10, 11, 15, 20, 22, 33, 40); in 1/22 cases, *BCR::ABL1* and *JAK2* showed an opposite growth, but in this case, under HU, *JAK2* decreased and *BCR::ABL1* remained positive (23). Finally, in 1/24 cases, *JAK2* and *BCR::ABL1* showed a similar growth as both decreased; however *JAK2* decrease was achieved with a combined TKI plus HU treatment (12).

### 4.14 Group 3: treatment and outcome

TKI treatment was administered in 21/23 cases with available therapy data; among these cases, TKI was the only therapy in 3/21 cases (1 PMF, 2 MPN, NOS) (10, 15, 29) and in 1/3 bortezomib and radiotherapy were also administered due to concomitant multiple myeloma (29); TKI and phlebotomy in 2/21 (PV) (11, 39); TKI and HU were administered in 5/21 cases (4 PMF, 1 ET) (7, 12, 14, 36, 40) with the adjunct of ASA in 1/5 (ET) (36); TKI, IFN and phlebotomy in 1/21 (PV) (22); TKI and IFN in 1/21 (PPV-MF) (37); TKI and lenalidomide in 1/21 (33); TKI, hydroxycarbamide and ASA in 1/21 (PMF) (20); TKI and ASA in 2/21 (ET) (20, 36); TKI, HU and ruxolitinib in 1/21 (PMF) (39); TKI, IFN and HU in 1/21 (ET) (34); TKI, anagrelide and HU in 1/21 (ET) (39); TKI and allo-HSCT in 1/21 (PMF) (39) and TKI, IFN, HU and allo-SCT in 1/21 (PMF) (44). In 1/23 cases (ET), HU and ASA without TKI were administered (23) and 1/23 (PPV-MF), HU and ruxolitinib without TKI (37). DMR of CML was reported in seven cases (7, 11, 15, 20, 34, 40, 44) and CCyR of CML in two cases (10, 20), but no further data on the outcome of these cases were reported. Data on outcome were available in 5/24 cases as follows: 2/5 cases were alive in hematological remission and three cases DOD. Of the two cases alive in hematological remission, one case (PV) received TKI and phlebotomy (39) and one (PMF) TKI and HU (12). Of the DOD cases, 1/3 (PMF) received TKI and allo-SCT (39), 1/3 (ET) received TKI, HU and anagrelide (39) and 1/3 (PMF) received TKI, ruxolitinib and HU (39).

## 5 Discussion

*JAK2* and *BCR::ABL1* are driver genomic alterations leading to different types of MPNs. Despite historically considered mutually exclusive driver mutant genes, the coexistence of *JAK2 V617* (or rarely *JAK2 exon 12*) and *BCR::ABL1* has been reported mainly as isolated case descriptions or small case series (4–53). In 2016, Martin-Cabrera et al. reported the incidence of 0.2% of *BCR::ABL1/JAK2 V617F* double-positive cases in a large cohort of 10.875 MPN cases (54). In 2018, Soderquist et al. estimated a frequency of 0.4% among 1570 MPNs tested for both genomic alterations (39). The previously reported higher frequency of 2.5% by Pieri et al. might be overestimated and likely related to the different cohorts selected in these studies as Pieri et al. evaluated only *BCR::ABL1*-positive cases, whereas Martin-Cabrera et al. and Soderquist et al. selected all MPN cases (21, 39, 54). Rare cases with coexistence of *CALR* and *BCR::ABL1* have been reported, but their discussion goes beyond the scope of the present paper (55, 56).

In our systematic review, we selected cases with clinically relevant dual driver mutations representing co-existent Ph-positive CML and Ph-negative MPN with *JAK2* mutation (4–53). It is well-known that very low level of *BCR::ABL1* transcripts may be found in some healthy individuals without clinical features of CML (57). Some Ph-negative MPNs, particularly ET, have been detected to have very low level of *BCR::ABL1* transcripts of no clinical and pathologic significance (38) and likewise, the presence of *JAK2* mutation, usually at low allelic burden, in the context of a CML may not change the clinical features of CML (58, 59). These situations likely represent examples of clonal hematopoiesis of indeterminate potential (CHIP) (38, 59).

In our systematic review, the majority of *BCR::ABL1/JAK2 V617F* double-positive cases fell into the group of Ph-negative MPN preceding CML (49.42%), followed by the group of CML and Ph-negative MPN occurring simultaneously (27.58%) and by the group of CML preceding Ph-negative MPN (22.98%). When Ph-negative MPN preceded CML, PV was the most commonly involved disease (20/43; 46.51%), followed by ET (12/43; 27.90%) and PMF (8/43; 18.60%). On the other hand, when CML preceded Ph-negative MPN, the most frequently detected was MPN, NOS (7/20; 35%) followed by PMF (6/20; 30%), PV (5/20; 25%) and ET (2/20; 10%). When Ph-negative MPN and CML occurred simultaneously, PMF was the most frequent disease (10/24; 41.66%) followed by ET (6/24; 25%) and by MPN, NOS (3/24; 12.5%) and PV (3/24; 12.5%) (see **Table 1**). Of note, MPNs preceding CML are more often non-fibrotic forms (PV, ET), whereas when CML is diagnosed before MPN, fibrosis is observed in most MPN cases at the time of diagnosis. It might be speculated that the onset of a MPN in the context of a CML under TKIs may be masked by the myelosuppressive effect of TKIs and, hence, only the fibrotic evolution gives sufficient force for the clinical and histological emergence of the MPN clone. On the other hand, the different distribution of MPNs may represent a real biological difference. More studies on large number of cases are needed for the understanding of this issue.

Hematologists and pathologists may face this confounding combination of genomic alterations, which can lead to clinical manifestations easily misinterpreted either as disease progression or resistance to treatment, hence, causing an inadequate management of the patient.

A modification in symptoms or laboratory data, during the course of Ph-negative MPNs, is commonly suspected to represent a sign of disease progression as Ph-negative MPNs may undergo myelofibrotic progression or transformation to acute leukemia or, more rarely, develop unusual types of progression with the occurrence of either monocytosis with the acquisition of (myelodysplastic/myeloproliferative) MDS/MPN-like features or leukocytosis with the acquisition of chronic neutrophilic leukemia–like features (1–3, 60). Likewise, a change in clinical manifestations in patients affected by CML is often interpreted as drug-resistance or a possible evolution to acute leukemia (1–3). However, when facing a change in the clinical course of either CML or Ph-negative MPN, hematologists and pathologists need to exclude even the possibility of a combination of *BCR::ABL1* translocation and *JAK2* mutation.

Clues to the likelihood of the coexistence of CML and Ph-negative MPN may be obtained from laboratory exams, modification in clinical symptoms as well from BM morphology. The appearance of constitutional symptoms, splenomegaly, PTL increase, RBC increase or cytopenia in patients with CML in MR should lead clinicians to rule out a coexisting Ph-negative MPN and mutations of *JAK2*, *MPL*, *CALR* should be evaluated. Likewise, in patients affected by a known Ph-negative MPN, an unexpected WBC or PTL increase or a change in symptoms should alert the clinician to exclude the emergence of CML. BM histology may reveal a shift of phenotype from CML to Ph-negative MPN or vice versa during treatment. It is well known that MK size and clustering may allow the distinction between the different types of MPNs with small-sized, hypo-lobate, non-clustering MKs more commonly found in CML and large, hyper-lobate clustering forms more typical of Ph-negative MPNs. The appearance of large hyper-lobate and clustering MKs with or without fibrosis as well as the identification of MKs with hybrid features (both small-sized, hypo-lobate and large-sized hyper-lobate forms) in the marrow of patients with a known CML should prompt testing for mutated *JAK2/CALR/MPL*. On the other hand, patients with a known Ph-negative MPN showing BM histological features reminiscent of CML should undergo cytogenetic and molecular testing to identify Ph chromosome and *BCR::ABL1* transcripts. Routinely performed BM biopsies during the course of Ph-negative and Ph-positive MPNs are essential to identify a possible shift of phenotype. The role of well-trained hematopathologists in the evaluation of BM biopsies is essential for the correct diagnosis; moreover, a strict integration between pathological features and molecular data is critical in particular in this setting of patients.

The clonal composition of MPNs harboring both *BRC::ABL1* and *JAK2 V617F* has been discussed in various reports, with some studies favoring the presence of two independent clones (8) and others supporting the hypothesis that the two genetic events occur in the same clone (15–17). Despite being rather evident that each of the two theories may be possible, the presence of two independent clones seems to represent the more common event. Interestingly, we observed an opposite growth of *BCR::ABL1* and *JAK2* under TKI treatment, with *BCR::ABL1* decrease and *JAK2* increase in the majority of patients of our systematic review (81.8% in concomitant CML and Ph-negative MPN; 65% in CML preceding Ph-negative MPN; 41.86% in MPN preceding CML). The increase in *JAK2* mutation burden during Ph-positive clone suppression by TKI treatment favors the hypothesis of two independent clones. Unfortunately, data on outcome in this subset of patients carrying both *BCR::ABL1* and *JAK2* are often incomplete or unavailable; therefore, it is rather difficult to draw conclusions on the optimal treatment. Whether a TKI associated with JAK2-inhibitor represents an optimal therapeutic strategy requires further evaluation. In those patients with dual anomalies, CML seems to be rather easy to manage, with often a good response of *BCR::ABL1* burden to different types of TKIs. The JAK2-positive MPNs have been treated with various therapies including HU, IFN, anagrelide and JAK2-inhibitors such as ruxolitinib, but the paucity of follow-up data does not allow to understand which is the optimal management strategy. Promising results with allo-SCT have been recently reported (see **Table 3**, **Supplementary Materials**), although data on a larger number of cases are needed (46).

## Author contributions

MZa: Conceptualization, Methodology, Writing – original draft, Writing – review & editing. AB: Data curation, Methodology, Writing – original draft. FS: Data curation, Methodology, Writing – original draft. GB: Data curation, Methodology, Writing – original draft. VF: Data curation, Methodology, Writing – original draft. MZi: Data curation, Writing – original draft. AP: Data curation, Writing – original draft. GM: Data curation, Writing – original draft. CaC: Data curation, Writing – original draft. CeC: Data curation, Writing – original draft. MC: Data curation, Writing – original draft. PG: Data curation, Writing – original draft. FG: Data curation, Writing – original draft. DT: Data curation, Writing – original draft. PP: Data curation, Writing – original draft. RC: Data curation, Writing – original draft. NK: Data curation, Writing – original draft. LC: Data curation, Writing – original draft. AC: Data curation, Writing – original draft. GF: Formal analysis, Investigation, Writing – original draft. SA: Conceptualization, Investigation, Supervision, Writing – review & editing.
