# Supplementary material for: Co-occurrence of JAK2-V617 F mutation and BCR::ABL1 translocation in chronic myeloproliferative neoplasms: a potentially confounding genetic combination
Source: Front Oncol. 2024 Jan 12;13:1329298. doi: 10.3389/fonc.2023.1329298 (PMC10811046; doi:10.3389/fonc.2023.1329298)
Supplement: Supplementary file 1 [file Table_1.docx]

Table 1. Characteristics of patients with concomitant or sequential JAK2-positive MPNs and CML

| **Reference/**  **Year** | **Age/**  **Sex** | **Disease course** | **Clinical/**  **laboratory signs suggestive of a second disease** | **BM findings suggestive of a second coexistent disease** | **t(9;22)**  **(q34;q11)**  **Clone interaction**  **(*BCR::ABL1 JAK2)*** | **Therapy**  **Outcome** |
| --- | --- | --- | --- | --- | --- | --- |
| Kramer 2007 | 50/M | CML,  PMF 4 yrs later | At the time of PMF  PTL decrease LDH increase WBC increase with immature myeloid cells in PB splenomegaly | PMF in fibrotic phase  (PMF histology evident after CMR with I, but PMF likely present from initial CML diagnosis; at the time of CML, BM histology showed initial fibrosis) | t(9;22)  (q34;q11) present  *BCR::ABL1* and *JAK2 V617F* coexistent from the time of CML;  with I,  *BCR::ABL1* decreased and *JAK2V617F* remained + at a constant allele frequency  Clone interaction: opposite growth | Hydrox, then I with CCyR and DMR.  Outcome NA |
| Inami  2007 | 43/M | CML,  PV 6 yrs later | At the time of PV  RBC increase HCT > 50% | NA | t(9;22)  (q34;q11) present  *BCR::ABL1* and *JAK2 V617F* coexistent from the time of CML;  with I,  *BCR::ABL1* decreased and *JAK2 V617F* remained + at a constant  allele frequency  Clone interaction: opposite growth | IFN for 1yr;  then CML (AP) and  IFN plus HU for 5 yrs; after I approval, I administered with CCyR; phleb for HT increase.  Outcome NA |
| Mirza  2007 | 82/F | PV,  CML 16 yrs later | At the time of CML  shortness of breath  feeling unwell hepatomegaly  massive splenomegaly macrocytic anemia  PTL increase WBC increase with neutrophilic shift and basophilia | NP | t(9;22)  (q34;q11) present  *JAK2V617F* + at time of PV;  *BCR::ABL1*+ at time of CML  Clone interaction NA | Radioactive Phosphorus, Hy, A (good PV control); after CML, I+ HU.  Died for cerebral event 4 mo after CML |
| Mirza  2007 | 73/F | PV, CML 15 yrs later | At the time of CML  shortness of breath fatigue hepatomegaly massive splenomegaly  WBC increase with neutrophilic shift and basophilia | CML (AP) | t(9;22)  (q34;q11) present  *JAK2 V617F*+ at time of PV;  *BCR::ABL1*+ at time of CML  Clone interaction NA | Phleb + A (good PV control); after CML, I administered, then stopped for adverse events.  Outcome NA |
| Bornhauser  2007 | 66/M | CML+  PMF  coexistent | At the time *BCR-ABL1* disappeared with I, anemia and progressive splenomegaly | BM fibrosis | t(9;22)  (q34;q11) present  *BCR::ABL1* and *JAK2 V617F* coexistent from diagnosis;  with I,  *BCR::ABL1* decreased and *JAK2* remained +  Clone interaction: opposite growth | I with transient DMR;  After *JAK2* test available and *JAK2*+ identified,  switch from I to HU.  Outcome NA |
| Bocchia  2007 | 43/M | PV, CML 16 yrs later | At the time of CML  WBC increase  HCT decrease progressive splenomegaly  At the time of MMR,  HCT increase with concomitant *JAK2* increase | NA | t(9;22)  (q34;q11) present  *BCR::ABL1*+  *JAK2 V617F*+27% at the time of CML;  with I,  *BCR::ABL1* decreased  and *JAK2* increased 49% (with concomitant HCT increase)  Clone interaction: opposite growth | Phleb. for 16 yrs; I with CCyR and DMR;  Phleb. again due to HCT increase  Outcome NA |
| Hussein 2007 | 55/M | CML, PMF 2 yrs later | NA | Post-TKI therapy:  PMF histology:  Increase in large-sized MKs with clustering + fibrosis (at this time *BCR-ABL1* negative and *JAK2* increase) | t(9;22)  (q34;q11) present  *BCR::ABL1* and *JAK2 V617F* coexistent from the time of CML (*JAK2* low burden 5%);  with I, *BCR::ABL1* decreased and *JAK2* increased up to 23%  Clone interaction: opposite growth | I with DMR and CCyR  Outcome NA |
| Kim  2008 | 49/M | CML, PMF 2 yrs later | At the time of PMF  PTL increase | PMF with fibrosis grade 3 | t(9;22)  (q34;q11) present  *BCR::ABL1* and *JAK2 V617F* (77.3%)  coexistent from the time of CML;  with Das,  *BCR::ABL1* decreased and *JAK2*  slightly decreased  (>50%)  remaining positive  Clone interaction: similar growth | I with insufficient response, replaced with Das with DMR and CCyR  Outcome NA |
| Kim  2008 | 64/M | CML+  PMF  coexistent | NA | BM fibrosis | t(9;22)  (q34;q11) present  *BCR::ABL1* and *JAK2 V617F*  (61%)  coexistent at diagnosis;  with Nil,  *BCR::ABL1* decreased and *JAK2*+  constant  Clone interaction: opposite growth | I with insufficient response, replaced with Nil with CCyR  Outcome NA |
| Cambier  2008 | 64/M | CML+PV coexistent | High WBC with neutrophilia high Hb, high HCT EPO below normal level | NA | t(9;22)  (q34;q11) present  *BCR::ABL1* and *JAK2 V617F* coexistent at diagnosis;  with I,  *BCR::ABL1* decreased and *JAK2* increased  Clone interaction: opposite growth | Phleb and I with DMR  (I had no effect on HCT and patient required phleb.)  Outcome NA |
| De Mello  Conchon  2008 | 52/F | CML+  PMF  coexistent | High WBC with neutrophilia and basophilia high PTL | MKs hyperplasia and BM fibrosis | t(9;22)  (q34;q11) present  *BCR::ABL1* and JAK2 V617F coexistent at diagnosis;  with I,  *BCR::ABL1* decreased, whereas  *JAK2* decreased only with combined I+Hy treatment  Clone interaction: similar growth | HU, then I alone,  (I effective in reducing *BCR-ABL1*, but due to PTL increase, combined treatment I+HU).  Alive and in HR at 6 mo from diagnosis |
| Jallades  2008 | 58/M | PMF, CML 2 yrs later | High WBC with basophilia eosinophilia  PTL decrease  splenomegaly | NA | t(9;22)  (q34;q11) present  *JAK2 V617F*+ and *BCR::ABL1*-, at the time of PMF;  at the time of CML,  *BCR::ABL1*+ and *JAK2 V617F* +;  with combined Hy+I,  *BCR::ABL1* decreased and *JAK2* still+  Clone interaction:  Opposite growth | I led to normal WBC, followed by combined treatment  HU+I, due to PTL increase  Outcome NA |
| Pardini 2008 | 67/M | CML+  PMF coexistent | Despite partial CyR and MMR (9 mo after I) persistence of anemia PTL increase splenomegaly | Initially BM showed CML+ focal fibrosis;  after I, PMF histology evident | t(9;22)  (q34;q11) present  *BCR::ABL1* and *JAK2* coexistent.  Clone interaction NA | I with subsequent addition of HU (9 mo later). PTL count normalized with HU  Outcome NA |
| Hussein 2008 | 32/M | CML, JAK2+  MPN, NOS 4 mo later | NA | At CML onset small MKs  After CMR with I, BM histology showed clustering, large MKs  (Ph-negative MPN phenotype) | t(9;22)  (q34;q11) present  *BCR::ABL1*+  *JAK2V617F* -, at CML diagnosis;  after CML DMR,  *BCR::ABL1*-, J*AK2V617F*+ (21%);  I therapy was continued with *JAK2V617F* decrease (from 21% to 5-6%).  Clone interaction: similar growth | A switched to HU for splenomegaly WBC increase;  2 mo later A replaced HU (for allergic reaction and PTL increase) with no positive effect on clinical data, then I with DMR.  Outcome NA |
| Hussein 2008 | 58/F | CML, PMF 1 yr later | Mild WBC increase  Splenomegaly  High LDH | PMF | t(9;22)  (q34;q11) present  Clone interaction NA | HU, then IFN.  Outcome NA |
| Hussein 2008 | 63/M | PV, CML 15 yr later | WBC increase | CML (CP) | t(9;22)  (q34;q11) present  *JAK2V617F*+ 62%  *BCR::ABL1*-, at the time of PV;  at the time of CML, *BCR::ABL1*+ *JAK2V617F*+75%;  with I, *BCR::ABL1*- and *JAK2*+ (51%); after IFN, *JAK2*+ (53%) and progression to MF; progression to AML with *JAK2*+ (90%) and *BCR-ABL1-.*  Clone interaction:  Opposite growth | I with DMR, but no effect on JAK2 clone; IFN with no effect on JAK2 clone and severe MF; 34 mo after CML, evolution to AML with death. |
| Hussein 2008 | 45/M | CML+  JAK2+  MPN  coexistent | WBC increase | Hybrid features with  CML histology+ clustering large MKs | t(9;22)  (q34;q11) NR  *BCR::ABL1*+  *JAK2V617F*+ 43%  at diagnosis;  with I, *BCR::ABL1* decrease and *JAK2*+  Clone interaction: opposite growth | I with DMR and *JAK2* still detectable  Outcome NA |
| Pingali  2009 | 39/M | PV, CML 18 yrs later | WBC increase anemia fatigue splenomegaly at CML occurrence.  After CMR with I, Hb increase with PV recurrence; then anemia and CML recurrence | CML (CP)  NA BM histology in other phases of disease | t(9;22)  (q34;q11) present  *BCR::ABL1*+  *JAK2 V617F*- at CML diagnosis;  after CML DMR, at PV recurrence *JAK2 V617F*+.  Clone interaction: opposite growth | Phleb for PV.  I with CCyR and DMR, then phleb. again for PV recurrence.  7 mo after stopping I, CML recurrence  Das, then Nil.  Outcome NA |
| Bee  2010 | 60/M | PV,  CML 1 yr later | Anemia  WBC increase  basophilia  leukoerythroblastosis  massive splenomegaly | CML (CP) | t(9;22)  (q34;q11) present  *JAK2 V617F* + at initial PV diagnosis, but transiently absent at CML diagnosis (when high *BCR::ABL1*) and increased again at CML CCyR, with PV recurrence  Clone interaction:  Opposite growth | Phleb for PV; I with CCyR and DMR; despite CCyR, Hb and WBC increase with PV recurrence; combined therapy: I+HU with good control of disease |
| Tefferi  2010 | 67/M | CML, PV 10 yrs later | PTL increase Hb increase WBC increase | PV | t(9;22)  (q34;q11) NR  *BCR::ABL1*+ (98.2%) and *JAK2 V617F*-, at CML diagnosis; at CML CCyR  *JAK2 V617F*+ (with low allele burden 1%) and not clinical evidence of PV. Slow and slight increase of *JAK2 V617* allele burden up to 6% at PV appearance  Clone interaction: opposite growth | IFN, cytosine arabinoside, HU and then I with CCyR  Outcome NA |
| Caocci  2010 | 70/M | CML, JAK2+  MPN,NOS 7 yrs later | Mild WBC increase | NA | t(9;22)  (q34;q11) present  *JAK2 V617F* NA  *BCR::ABL1*+ at CML diagnosis;  7 yrs after initial diagnosis, *BCR::ABL1*+ and  *JAK2 V617F*+;  at the time of CML CCyR, *JAK2* still +  Clone interaction: opposite growth | IFN with good clinical condition, but cytogenetic data NA; after 7 yrs, I with persistent CCyR  Outcome NA |
| Veronese  2010 | 82/F | CML+ PMF coexistent | PTL increase splenomegaly despite CML MMR | BM biopsy performed 12 mo after I:  MK increase and fibrosis | t(9;22)  (q34;q11) present  *BCR::ABL1*+  *JAK2V617F*+ (initially at low burden 0.5%) at diagnosis; with I,  *BCR::ABL1*- *JAK2*+ 92%  Clone interaction:  opposite growth | Hydrox, I, ASA  with DMR of CML at 36 mo and normal PTL count |
| Veronese  2010 | 62/M | CML+ ET coexistent | PTL increase | CML (CP) | t(9;22)  (q34;q11) present  *BCR::ABL1*+  *JAK2V617F*+ (initially at low burden: 0.2%) at diagnosis; with I,  *BCR::ABL1*- *JAK2*+ 9%  Clone interaction:  opposite growth | I, ASA CCyR, but persistent high PTL count |
| Pieri  2011 | 82/F | PV, CML 10 yrs later | WBC increase | CML (CP) | t(9;22)  (q34;q11) present  *BCR::ABL1*+  *JAK2 V617F*+ low allele burden (1-2%) at CML diagnosis;  under I and Das,  *BCR-ABL1* decrease  *JAK2* increase 61%  Clone interaction:  Opposite growth | Pipobroman, Hy for PV, then I and Das with hematologic response  Outcome NA |
| Hummel  2012 | 70/M | CML+ PV  coexistent | WBC increase with neutrophilia basophilia eosinophilia splenomegaly at diagnosis;  after I, WBC decrease with progressive Hb and HCT increase | High cellularity myeloid and MK hyperplasia increase of clustering MKs; grade 2 fibrosis  (MF- like histology) | t(9;22)  (q34;q11) present  *BCR::ABL1* and *JAK2V617*F 44.9% coexistent at diagnosis;  *JAK2* increase with the emergence of PV-like phenotype  Clone interaction: opposite growth | I with WBC decrease. While CML parameters improved, Hb and HCT increased, then phleb and IFN  Outcome NA |
| Hummel  2012 | 63/M | ET, CML with fibrosis 2 yrs later | 2 yrs after ET, WBC and PTL increase | At the time of CML,  PET-MF: high cellularity with increased myeloid: erythroid ratio, increase of clustering atypical MKs; grade 3 fibrosis with osteosclerosis) | t(9;22)  (q34;q11) present  *BCR::ABL1*- and *JAK2 V617F*+ at the time of ET;  at the time of CML,  *JAK2 V617F*+ (80.8%) and *BCR::ABL1*+  Clone interaction NA | I, Das, then I and HU, then IFN plus I and HU. Normal blood parameters with IFN. Under evaluation for HSCT |
| Hummel  2012 | 70/M | CML, JAK2+  MPN, NOS 6 mo later | Despite good molecular response with I, WBC increase | At the time of CML, high cellularity, increased myeloid: erythroid ratio, increase of MKs with dispersed hypo-lobate forms, but even many large forms with bulbous nuclei, MK clustering occasionally.  At the time of *BCR-ABL1* decrease with TKI and *JAK2* detection,  high cellularity, myeloid hyperplasia  atypical, large, bulbous MKs; grade 2 fibrosis  (Ph-negative MPN phenotype) | t(9;17;22)  (q34;p12;q11)  *BCR::ABL1*+ at the time of CML; with TKIs,  *BCR::ABL1* decrease and *JAK2 V617*+ 8.7%  Clone interaction:  Opposite growth | I, Das, HU, Nil, spleen RT.  Died for pneumonia 76 mo after initial diagnosis |
| Xiao  2012 | 77/M | ET, then PET-MF, CML 12 yrs later | WBC increase with basophilia anemia PTL increase splenomegaly | At the time of CML, PET-MF histology (MK hyperplasia  Grade 3 fibrosis) | t(9;22)  (q34;q11) present  *JAK2 V617F*+ and *BCR::ABL1*+ 90.9% at CML diagnosis  Clone interaction  NA | HU and ASA irregularly; then Hy, Th ASA, (patient refused further therapy)  Outcome NA |
| Xiao  2012 | 46/F | ET and CML coexistent | PTL increase anemia WBC increase with neutrophilia basophilia | MK hyperplasia | t(9;22)  (q34;q11) present  *JAK2 V617*+  *BCR::ABL1*+ at diagnosis;  after Hy, *JAK2-* and *BCR::ABL1*+  Clone interaction: opposite growth | ASA and HU  (patient refused I).  At present good control of blood parameters and BCR-ABL1+ |
| Inokuchi  2012 | 43/M | CML, PV 5 yrs later | RBC increase  HCT increase | BM NA | t(9;22)(q34;q11) present  *JAK2 V617F* 20% and *BCR::ABL1* coexistent at CML diagnosis;  with Das, *JAK2-*  *BCR::ABL1*-  Clone interaction: similar growth | IFN, HU for 5 yrs, then I with CCyR  Phleb.  13 yrs later, CML relapse, Das with good response and negativity of both genomic markers |
| Lee  2013 | 53/F | CML, ET 10 yrs later | Despite CCyR, WBC and PTL increase | Increased MKs | t(9;22)  (q34;q11)  At the time of CML, BCR::ABL1+ and JAK2 V617F -,  JAK2 + 10 yrs later at the time of CCyR  Clone interaction: opposite growth | HU, IFN, I. CCyR and DMR with I.  When JAK2 became +, I+A+HU.  At 13 yrs from CML diagnosis, good hematological parameters, CCyR and MMR and JAK2 still + |
| Lee  2013 | 60/F | CML, ET 2 yrs later | Despite CCyR, PTL increase | NA | t(9;22)  (q34;q11)  *BCR::ABL1*+ and *JAK2 V617* -, at CML diagnosis;  2 yrs later at the time of CCyR *JAK2* +  Clone interaction: opposite growth | I with CCyR and DMR; when *JAK2* became +, I+A; then Nil+A. At 3 yrs from CML diagnosis, good clinical conditions, CCyR and DMR, *JAK2* still + |
| Pastore  2013 | 42/F | CML, JAK2+ MPN, NOS, 8 yrs later | PTL and LDH increase despite MMR of CML | NA | t(9;22)  (q34;q11)  *BCR::ABL1*+ 98%, *JAK2V617*F+ (at low burden) at CML diagnosis,  with I and DAS, *BCR::ABL1* decrease, *JAK2* increase  Clone interaction: opposite growth | HU, I, Das with CCyR and DMR |
| Ursuleac  2013 | 61/M | PV, CML 7 yrs later | WBC increase with neutrophilia and basophilia anemia low PTL  High LDH  Dyspnea on exertion  hepatosplenomegaly | CML (CP) | t(9;22)  (q34;q11) present  *BCR::ABL1*+ (100%) and *JAK2 V617F* -, at CML diagnosis;  2 yrs after CML, switch to PV-phenotype  *JAK2 V617F*+  and *BCR::ABL1*-  Clone interaction:  Opposite growth | Phleb, HU for PV; I with DMR and CCyR.  When the PV-phenotype re-presented (HCT increase) phleb. added to I. Good control of disease |
| Yamada  2014 | 67/M | PMF, CML 3 yrs later | WBC increase splenomegaly | At the time of PMF,  MKs increase and grade 3 fibrosis;  at the time of CML, identical BM histology with PMF features | t(9;22)  (q34;q11) present  *JAK2 V617F*+, *BCR::ABL1* NA, at PMF diagnosis;  at the time of CML,  *JAK2 V617F*+ BCR::ABL1+ 90%;  with TKIs, *BCR::ABL1*- and *JAK2 V617F*+  Clone interaction:  Opposite growth | Nil, Das with DMR  Outcome NA |
| Maerki  2014 | 77/M | Patient with MM and coexistent CML and JAK2+  MPN | anemia | MM+  Hybrid features with clustering of hypo-lobate atypical MKs+  megaloblastic change | t(9;22)  (q34;q11)  *JAK2 V617F* and *BCR::ABL1* coexistent  Clone interaction NA | RT Bortez. and TKI  Outcome NA |
| Quin  2014 | 25/F | ET and CML coexistent | Pregnant patient  high PTL and high WBC | NA | t(9;22)  (q34;q11)  *BCR::ABL1*+ 161%  *JAK2 V617F*+  coexistent  Clone interaction NA | NA |
| Zhou  2015 | 55/F | PV, CML 10 yrs later | WBC increase anemia leukoerythroblastosis splenomegaly  weight loss fatigue | CML with moderate fibrosis | t(9;22)  (q34;q11) present  *BCR::ABL1*+ 92.5% *JAK2 V617F*+ (with low allele burden 6%) at CML diagnosis;  with Das, *BCR::ABL1* decrease to 4%, but WBC and PTL increase as well as *JAK2 V617F* increase to 83%; at the time of CCyR,  *BCR::ABL1* - *JAK2 V617F*+ increased to 96%  Clone interaction:  Opposite growth | ASA, HU, A for PV, Das.  With Das, despite *BCR-ABL1* decrease 8%, WBC and PTL increase with *JAK2 V617F* increase; then HU and A with no results, followed by Das+ Rux. CCyR 3 yrs after combined therapy |
| Chen  2015 | 78/F | ET, CML 10 yrs later | WBC increase splenomegaly | CML (CP) | t(9;22)  (q34;q11) present  *JAK2 V617F*+ at ET diagnosis, *BCR::ABL1+* and *JAK2 +* at CML diagnosis  Clone interaction: NA | HU, A, I, Nil, Pon with DMR; then CML (BP) 4 yrs after CML diagnosis and combined therapy: Pon+ cytarabine+rux  Poor response to therapy |
| Wang  2015 | 51/M | CML and PMF coexistent | WBC increase  neutrophilia  basophilia  leukoerythroblastosis  high LDH | CML+PMF features | t(9;22)  (q34;q11) present  *BCR::ABL1*+ 38.52%  *JAK2V617F*+ 51.3% at diagnosis; with I, *BCR::ABL1*-  *JAK2V617F*+ 39.8%  Clone interaction: opposite growth | I, lenalidomide  Outcome NA |
| Borgia Barbosa Pagnano  2016 | 73/M | CML and ET coexistent | Despite CCyR and MMR, high PTL | Pre-TKI therapy:  CML histology  Post-TKI therapy: ET histology | t(9;22)  (q34;q11) present  *BCR::ABL1*+ at CML diagnosis; *JAK2V617F*+ at ET diagnosis  Clone interaction: NA | IFN, then I with  CCyR and DMR, but persistent high PTL count; then I+HU; DMR at 10 yrs after CML diagnosis  Outcome NA |
| Paz  2016 | 45/F | PV, CML 16 yrs later | WBC increase basophilia | NA | t(9;22)  (q34;q11) present  *JAK2V617F*+ at PV diagnosis and constantly+ during the course of PV and CML;  *BCR::ABL1*+ at CML diagnosis  Clone interaction: similar growth | Phleb, pipobroman, HU, spleen RT, IFN, Rux, I. Death for gastric cancer |
| Darling  2017 | 69/F | CML and JAK2+  MPN (probable ET) coexistent | Persistent high PTL despite I | Panmyelosis and MK clustering | t(9;22)  (q34;q11) NR  *BCR::ABL1*+  *JAK2 V617F*+  coexistent  Clone interaction NA | I, then I stopped and HU+ASA; *BCR-ABL1*+ again and I with HU  Outcome NA |
| Darling  2017 | 57/F | CML and ET coexistent | High WBC with neutrophilia and basophilia High PTL | High cellularity  myeloid hyperplasia increase of clustering MKs  (mixed features: CML and ET) | t(9;22)  (q34;q11)  *BCR::ABL1+* (38.51%) and *JAK2 V617F*+ coexistent  Clone interaction NA | I and ASA  Outcome NA |
| Kandarpa 2017 | 63/M | PET-MF, CML 2 yrs later | WBC and PTL increase | Partial BM data:  Grade 3 fibrosis | t(9;22)  (q34;q11) NR  *JAK2 V617F* and *BCR::ABL1* coexistent at CML diagnosis  Clone interaction NA | HU, IFN, I |
| Kandarpa  2017 | 59/F | ET, CML 13 yrs later | anemia | Partial BM data:  Grade 3 fibrosis | t(9;22)  (q34;q11) NR  *JAK2 V617F* and *BCR::ABL1* coexistent at CML diagnosis  Clone interaction NA | HU, I, Das |
| Kandarpa  2017 | 68/F | PPV-MF, CML 15 yrs later | Anemia low PTL | Partial BM data:  Grade 3 fibrosis | t(9;22)  (q34;q11) NR  *JAK2 V617F* and *BCR::ABL1* coexistent at CML diagnosis  Clone interaction NA | Rux, TKI |
| Kandarpa  2017 | 56/F | PPV-MF+CML coexistent | Anemia splenomegaly | Partial BM data:  Grade 1-2 fibrosis | t(9;22)  (q34;q11) NR  *JAK2* exon 12 and *BCR::ABL1* coexistent at CML diagnosis  Clone interaction NA | Rux, HU |
| Kandarpa  2017 | 70/M | CML, PPV-MF coexistent | WBC increase splenomegaly | Partial BM data:  Grade 2 fibrosis | t(9;22)  (q34;q11)  *JAK2 V617F* and *BCR::ABL1* coexistent at CML diagnosis  Clone interaction NA | IFN, I |
| Boddu  2018 | 61/M | CML, JAK2+ MPN, NOS 6 yrs later | PTL increase  (despite CMR) | Increase of clustering MKs | t(9;22)  (q34;q11)  *JAK2 V617F* NP,  *BCR::ABL1*+ 33 % at CML diagnosis;  with I,  *BCR::ABL1*-, *JAK2 V617F*+ 24%.  Clone interaction:  Opposite growth | I with DMR, then HU with good PTL control  Outcome NA |
| Boddu  2018 | 80/M | CML, JAK2+ MPN, NOS 5 mo later | PTL increase and WBC increase with neutrophilia | High cellularity Increase of large mono-lobate and clustering MKs | t(9;22)(q34;q11)  *BCR::ABL1*+  *JAK2 V617F* NP at CML diagnosis; 5 mo after CCyR,  *BCR::ABL1*+ 8%  *JAK2 V617F*+ 28%;  then *JAK2V617- BCR::ABL1* NP  Clone interaction:  Opposite growth | Nil with CHR and CCyR for 5 mo; then for CML relapse and JAK2+MPN Nil+HU. Died with CML BP 1 yr later |
| Boddu  2018 | 49/F | PV, then PPV-MF, CML 9 yrs later | WBC increase with neutrophilia | At CML diagnosis:  CML (CP) histology  (Myeloid hyperplasia an  MKs shifted from clusters of large-sized with hyper-  lobate nuclei at the time of PV to small hypo-lobate MKs | t(9;22)(q34;q11) present  *BCR::ABL1- JAK2 V617F +* 24% at PPV-MF diagnosis; at the time of CML, *BCR::ABL1* + 100% and *JAK2 V617F*+ 26%  Clone interaction  NA | IFN and Rux for PPV-MF, then I for CML with suboptimal response and possible switch to Nil |
| Boddu 2018 | 71/F | ET, then PET-MF, CML 3 yrs later | WBC increase with neutrophilia basophilia anemia low PTL | NP | t(9;22)(q34;q11) NR  *JAK2V617F*+21% *BCR::ABL1* - at PET-MF diagnosis;  at CML diagnosis,  *BCR::ABL1*+  100%  *JAK2V617F*+ 6%; with TKIs, *BCR::ABL1*- and *JAK2V617F* increase (55.8%)  Clone interaction:  opposite growth | Rux for PET-MF; then I followed by Bos with  DMR and no symptoms without therapy for PET-MF |
| Boddu 2018 | 87/F | ET, CML 11 yrs later | NA | At the time of CML,  PET-MF+ CML histology (MKs with hybrid features)  High cellularity, clusters of large, atypical, hypo-lobate and hyper-lobate MKs Fibrosis grade 2 | t(9;22)  (q34;q11) present  JAK2 V617F +18.3% and *BCR::ABL1*+ 100% at CML diagnosis  Clone interaction NA | A with good ET control, I, then Pon with CCyR. Pon stopped for side effects and CML progression to AP; then Rux+  Cytarabine+  Pon.  CML progression to BP with death |
| Soderquist  2018 | 48/F | PMF, CML 10 yrs and 9 mo later | WBC and PTL increase anemia | CML (CP) | t(9;22)  (q34;q11) present  *JAK2 V617F+ BCR::ABL1*- at PMF diagnosis; at the time of CML, *BCR::ABL1*+ 94%  *JAK2 V617F*+ 10-50%; at the time of DMR,  *BCR::ABL1* -  *JAK2 V617F*+ 78%  Clone interaction:  Opposite growth | No therapy at PMF diagnosis; then  Nil HU Rux  MMR  AWD (PMF) |
| Soderquist  2018 | 66/F | ET, then PET-MF and CML 3 yrs and 9 mo later | WBC increase  anemia low PTL | PET-MF+ CML | t(9;22)  (q34;q11) present  *JAK2 V617F*+  *BCR::ABL1*- at ET diagnosis; at the time of CML,  *BCR::ABL1*+  t(9;22) 80% *JAK2 V617F*+ 24%; no further evaluation  Clone interaction NA | HU, Rux for PET-MF; then I, Bos, Rux.  No MMR AWD (MF) |
| Soderquist  2018 | 48/F | PV, then PPV-MF and CML 4 yr and 10 mo later | WBC increase  Low PTL | PPV-MF+CML | t(9;22)  (q34;q11) present  *BCR::ABL1*-  *JAK2 V617F*+ at PV diagnosis; at the time of CML  *BCR::ABL1*+ t(9;22) 100%  *JAK2V617F* + 24.4%;  no further evaluation  Clone interaction  NA | Phleb. HU IFN for PV; then I IFN Rux  No MMR  AWD (MF) |
| Soderquist  2018 | 60/F | PV, then PPV-MF and CML 107 mo later | WBC increase anemia low PTL | PPV-MF+ CML | t(9;22)  (q34;q11) present  *BCR::ABL1-*  *JAK2 V617F*+ at PV diagnosis; at the time of CML,  *BCR::ABL1*+ 63%  t(9;22) 80%  *JAK2* >50%;  no further evaluation  Clone interaction NA | HU Th Rux for PV; then I Hy Th Rux  No MMR  DOD |
| Soderquist  2018 | 76/M | PV, CML (AP) 73 mo later | WBC increase anemia low PTL | CML (AP) | t(9;22)  (q34;q11) present  *BCR::ABL1-*  *JAK2* NP at PV diagnosis;  at the time of CML,  *BCR::ABL1*+ 82%  J*AK2 V617F*+ <10%; with I,  *BCR::ABL1*+  *JAK2 V617F* +<10%  Clone interaction  Similar growth | HU for PV, then I Hy  No MMR  DOD (CML AP) |
| Soderquist  2018 | 49/F | CML, then JAK2+  MPN, NOS 109 mo later | PTL increase | MPN, NOS | t(9;22)  (q34;q11) NR *BCR::ABL1*+ *JAK2* NP at CML diagnosis;  at the time of Ph-negative MPN, *BCR::ABL1*-  *JAK2* NP; at FU *JAK2 V617F*+ (28%)  Clone interaction: NA | I IFN for CML; then I Das  No MMR  AWD (MF) |
| Soderquist  2018 | 70/M | Initial diagnosis NA; then CML and PMF coexistent | WBC increase anemia | CML and PMF | t(9;22)  (q34;q11)  At the time of CML and PMF, *BCR::ABL1*+  *JAK2 V617F*+;  at FU *JAK2V617F* 7.6%  Clone interaction NA | I Nil Das HU Rux  No MMR  DOD (MF) |
| Soderquist  2018 | 68/M | Initial diagnosis NA, then CML | WBC and PTL increase | CML (CP) | t(9;22)  (q34;q11) present  *BCR::ABL1*+  *JAK2V617F*+ <10% at CML diagnosis; at FU  *JAK2 V617F*+ >25%  Clone interaction:  Opposite growth | I Das A  MMR  Alive in remission |
| Soderquist  2018 | 49/M | Initial diagnosis NA, then CML and PMF | WBC increase anemia | CML and PMF | t(9;22)  (q34;q11)  At the time of CML and PMF, *BCR::ABL1*+  *JAK2 V617F*+; at FU  *JAK2 V617F*<1  Clone interaction NA | I Das Allo-SCT  DOD (CML-AP) |
| Soderquist  2018 | 81/F | Initial diagnosis NA, then CML and ET | WBC increase | CML and ET | t(9;22)  (q34;q11)  At the time of CML and ET,  *BCR::ABL1+*  *JAK2 V617F+* 18.3%; at FU  *JAK2 V617F* NP  Clone interaction NA | I Nil A HU  No MMR  DOD (MF CML-BP) |
| Soderquist  2018 | 73/F | Initial diagnosis NA, then CML and PV | WBC increase | CML and PV | t(9;22)  (q34;q11)  At the time of CML and PV,  *BCR::ABL1*+ 5%  t(9;22) 10%  *JAK2 V617F*+; at FU  *JAK2 V617F*+  Clone interaction NA | I and Phleb  No MMR  Alive in remission |
| Bader  2019 | 75/M | CML and PMF coexistent | Weight loss night sweats pruritus hepatosplenomegaly  WBC increase with neutrophilia  PTL increase | BM histology only after I with CMR:  High cellularity  Myeloid hyperplasia, Increase of  atypical MKs  Grade 2 fibrosis | t(9;22)  (q34;q11) NR  BCR::ABL1+ and JAK2 V617F+ at diagnosis;  with I,  *BCR::ABL1-*  *JAK2 V617F*+  Clone interaction: opposite growth | I, HU  DMR  Outcome NA |
| Swaminathan  2019 | 67/M | PV,  CML 3 yrs later | WBC increase with neutrophilia eosinophilia basophilia | CML with mild fibrosis | t(9;22)  (q34;q11)  *JAK2* exon 12+ (<5%) at the time of PV; at the time of CML  *BCR::ABL1*+  (72%) and *JAK2* exon 12+ (<5%); with I,  *BCR::ABL1*- and *JAK2* exon12+27%  Clone interaction:  Opposite growth | Phleb., ASA and HU; then I  CCyR and DMR 1 yr later |
| Tirrò  2019 | 61/M | ET, CML 3 yrs later | WBC increase | CML (CP) | t(9;22)  (q34;q11) present  *JAK2 V617F*+ 25% at ET diagnosis;  at the time of CML, *BCR::ABL1+*  *JAK2*+ with increase allele burden 50%  Clone interaction NA | ASA, HU, then Nil, Das, I with MMR; due to toxicity, TKI temporarily suspended; then IFN+HU added to I to control PTL increase; then A+I.  Due to lung cancer, I stopped  Outcome NA |
| Lorenzo  2020 | 70/F | PV, CML 6 yrs later | WBC increase  leukoerythroblastosis  splenomegaly | CML (CP) | t(9;22)  (q34;q11) NR  *JAK2 V617F*+ at PV diagnosis; at the time of CML,  *BCR::ABL1*+99% *JAK2 V617F* -; after I, *BCR::ABL1-*  *JAK2 V617F*+  (HCT increase)  Clone interaction: opposite growth | Phleb. ASA HU; then I  and Das with DMR, Phleb for HCT increase  Outcome NA |
| Yue  2020 | 50/F | PMF and CML coexistent | WBC increase with neutrophilia PTL increase  Anemia  High LDH  Splenomegaly | PMF histology  Severe fibrosis and increase of clustering MKs | t(9;22)  (q34;q11)  *BCR::ABL1*+ 85% and *JAK2* exon 18 9.27%  Clone interaction NA | Progression to AML  IFN HU  CT (Idarubicin cytarabine), Das allo-SCT  DMR  Outcome NA |
| Zhao  2021 | 46/M | PV, CML  9 yrs later | WBC increase, with left-shifted neutrophilia basophilia eosinophilia  leukoerythroblastosis  anemia  low PTL count splenomegaly | At the time of PV, hyperplastic hematopoiesis with increase of large lobate MKs; at CML diagnosis,  PPV-MF+CML with  hybrid MKs  (High cellularity  Myeloid hyperplasia  Increase of  large hypo-lobate MKs (hybrid form between PV and CML)  fibrosis | t(9;22)  (q34;q11) present  *BCR::ABL1- JAK2 V617 F*+ (>50%) at PV diagnosis;  at the time of CML,  *BCR::ABL1*+ 48% and *JAK2 V617F*+ 84%  TP53+ (40%)  del(20q);  in the disease course, increase of *JAK2* 93.5% and *BCR::ABL1* 89.1%  Clone interaction:  similar growth | Phleb. HU Rux  Spleen RT, then allo-SCT; alive 4 mo after allo-SCT  with undetectable  genomic markers |
| Sorà  2021 | 24/F | ET, then PET-MF, CML after 17 yrs | Persistent WBC increase | PET-MF | t(9;22)  (q34;q11) present  *JAK2 V617F*+  *BCR::ABL1*- at ET diagnosis;  at the time of CML,  *BCR::ABL1*+ and *JAK2* NR; before allo-SCT, MMR of CML and *JAK2* + (58%)  Clone interaction:  Opposite growth | IFN with benefit; then phleb and A.  for increased HCT; then I+A with MMR. A stopped because of anemia and HU introduced for PTL increase. Blastic progression.  Allo-SCT.  Alive in complete remission of both diseases at 24 mo after allo-SCT |
| Sorà  2021 | 58/F | CML, PMF 2yrs later | Despite MMR of CML with Nil, high PTL persisted | PMF with severe fibrosis | t(9;22)  (q34;q11) NR  *BCR::ABL1*+ at CML diagnosis; at the time of persistent high PTL despite MMR of CML, *JAK2 V617F+* (>50%)  Clone interaction:  Opposite growth | Nil with MMR; then Rux, finally allo-SCT.  Alive in complete remission at 4 mo after allo-SCT |
| De Bruyne  2021 | 59/F | ET, CML 2 yrs later | WBC increase  eosinophilia  basophilia | CML+fibrosis | t(9;22)  (q34;q11) NR  *BCR::ABL1*-  *JAK2 V617F*+3.6% at the time of ET diagnosis; at the time of CML, *BCR::ABL1+* *JAK2*-  Clone interaction: opposite growth | ASA, HU, Das with complete hematologic remission |
| Zhao  2022 | 50/F | PV, CML 13 yrs later | WBC increase with left-shifted neutrophilia basophilia  leukoerythroblastosis eosinophilia splenomegaly  fatigue decreased appetite weight loss | At the time of PV, slightly high cellularity Increase of large, lobate MKs;  at the time of  CML, PPV-MF histology:  (Myeloid hyperplasia Increase of MKs, grade 3 fibrosis) | t(9;22)  (q34;q11) NP  *JAK2 V617F*+  *BCR::ABL1*- at PV diagnosis; at the time of CML, *BCR::ABL1*+  *JAK2 V617F*+  (>50%)  Clone interaction NA | Phleb. HU Rux Nil  Died of sepsis, DIC organ failure 4 mo later |
| Zhao  2022 | 77/M | ET, CML 10 yrs later | WBC increase with left shifted neutrophilia  basophilia  leukoerythroblatosis  PTL increase  LDH increase | At the time of ET, Slightly high cellularity Increase of large lobate MKs;  At the time of CML,  Histology of CML+ hybrid MKs  (High cellularity  Myeloid hyperplasia, Increase of both small hypo-lobate and large lobate MKs) CLL-like clonal B cells (9%) | t(9;22)  (q34;q11) present  *BCR::ABL1- JAK2V617F*+ at ET diagnosis; at the time of CML,  *BCR::ABL1+*  *JAK2 V617F*+18%  Clone interaction NA | HU I Pon, then HU stopped and A added  No response to therapy  Poor condition at 42 mo |
| Zhao  2022 | 48/F | PMF, CML 4 yrs later | Persistent WBC increase for 4 yrs before *BCR-ABL1* acquisition  left-shifted neutrophilia  basophilia  eosinophilia  splenomegaly | At the time of PMF,  High cellularity  Myeloid hyperplasia Increase of large lobate MKs  Mild- moderate fibrosis;  at the time of CML,  CML+fibrosis  (High cellularity Myeloid hyperplasia  Increase of small hypo-lobate MKs  Severe fibrosis) | t(9;22)  (q34;q11)  *BCR::ABL1-*  *JAK2 V617F*+ at PMF diagnosis; at the time of CML, *BCR::ABL1*+ 100%  *JAK2 V617F*+  Clone interaction NA | HU, then stopped for side effects;  I, Nil Sun Pon Das Omacetaxine and then Rux with HU, splenectomy and allo-SCT  Alive with no detectable genomic markers, 49 mo after allo-SCT |
| Zhao  2022 | 58/M | PMF, CML 5 yrs later | Fatigue progressive splenomegaly  leukoerythroblastosis  with 12.6% blasts and basophilia | At the time of PMF, High cellularity  Increase of hyperchromatic lobate MKs  Moderate fibrosis  At the time of CML,  Histology of CML+fibrosis  High cellularity  Myeloid hyperplasia Increase of hypo-lobate MKs  Grade 3 fibrosis  10.3% blasts | t(9;22)  (q34;q11)  *BCR::ABL1- JAK2 V617F*+ at PMF diagnosis; at the time of CML,  *JAK2 V617F* NP  t(9;22)+  Clone interaction: NA | Rux, Nil; allo-SCT  Alive with no detectable genomic markers, 17 mo after CML |
| Zhao  2022 | 76/M | CML, PMF 1 yr later | Splenomegaly  WBC increase anemia low PTL no basophilia | At the time of CML, High cellularity, Myeloid hyperplasia  Increase of small hypo-lobate MKs;  at the time of PMF,  PMF histology  Increase of hyperchromatic lobate MKs Grade 3 fibrosis | t(9;22)  (q34;q11) NP  *BCR::ABL1*+ 94%  *JAK2 V617F*- at CML diagnosis;  at the time of PMF, *BCR::ABL1-*  *JAK2 V617F*+ 35%  Clone interaction: opposite growth | Nil  Rux, Nil HU; scarce response to therapy  Outcome NA |
| Zhang  2022 | 48/M | ET, then PET-MF,CML (AP) 10 yrs later | WBC increase with neutrophilia basophilia, 5% blasts  hepatosplenomegaly | CML with fibrosis | t(9;22)  (q34;q11) present  *JAK2 V617F+*  *BCR-ABL1*- at ET diagnosis;  at the time of CML,  *BCR::ABL1*+ 92%  *JAK2 V617F*+ 91.38%; after Fum+Rux  *BCR::ABL1* 0.24%  *JAK2*+ 92.54%  Clone interaction:  opposite growth | HU, then IFN for ET; then Rux for PET-MF; then I for CML, I suspended for intolerance, then  Fum+Rux.  RT and allo-SCT are under consideration |
| Ryu  2022 | 58/F | PMF, CML 7 yrs later | WBC increase basophilia anemia and circulating blasts  splenomegaly | CML (AP)  MKs in clusters+ fibrosis | t(9;22)  (q34;q11) present  *BCR::ABL1*- *JAK2V617*+ at PMF diagnosis; at CML diagnosis *BCR::ABL1*+ *JAK2*+  Clone interaction: NA | HU, Rux, I with scarce response to therapy |
| Hochman  2023 | 58/F | PV, CML 30 yrs later | Fatigue splenomegaly  WBC increase | CML (CP) | Variant t(9;14;22)  *BCR::ABL1*+  *JAK2 V617F*+ at CML diagnosis  Clone interaction NA | Phleb., then I  With CMR after 10 mo |
| Hochman  2023 | 44/F | PV, CML 14 yrs later | WBC increase | At the time of CML,  PPV-MF histology  (High cellularity, increased MKs with hyper-lobate nuclei  Moderate fibrosis) | t(9;22)  (q34;q11) present  *JAK2 V617F*+ >80% at PV diagnosis; at the time of CML, *BCR::ABL1*+ 78%  *JAK2 V617F*+ 94.31%;  with Nil,  *BCR::ABL1* decrease  *JAK2* + stable  Clone interaction:  Opposite growth | Phleb., then Rux; then Nil with dropping of *BCR-ABL1*  Outcome NA |
| Tosoni  2023 | 59/M | CML, PV 20 yrs later | High Hb High HCT Low EPO | NA | t(9;22)  (q34;q11) NR  *BCR::ABL1+* at CML diagnosis; *JAK2 V617F* + at low allelic burden (1.8) identified during TKIs, then *JAK2* at higher allelic burden (37%) at CML DMR and PV-phenotype emergence  Clone interaction:  Opposite growth | I, IFN, Das, Nil, Bos, Pon with DMR after 20 yrs; phleb. HU and ASA; good clinical conditions and blood parameters at 2 yrs after PV |
| Lapietra 2023 | 48/M | CML, PV 17 yrs later | High Hb high HCT | NA | t(9;22)  (q34;q11) present  *BCR::ABL1*+ *JAK2V617F*- at CML diagnosis; *JAK2V617F*+ 2 yrs after CML diagnosis and 1 yr after DMR, but PV symptoms only 17 yrs after CML diagnosis  Clone interaction:  Opposite growth | HU, IFN, I with DMR 1 yr after CML diagnosis;  then phleb+ASA+I  Good control of disease |
| Present report  Case 1 | 56/F | Pre-fibrotic PMF, CML 13 yrs later | WBC increase | At the time of CML:  CML (CP) histology  (High cellularity myeloid hyperplasia  Increase of small-sized MKs with scarce tendency to loose clustering).  At the time of CCyR and *JAK2* increase:  PMF histology  (High cellularity  myeloid hyperplasia increase of variably-sized MKs in clusters) | t(9;22)  (q34;q11) present  *BCR::ABL1-*  *JAK2 V617F*+ 83.98% at PMF diagnosis;  at the time of CML,  *BCR::ABL1*+ 99%  *JAK2*+ 21.48%  Despite CCyR with I,  *JAK2* increase  89.83%  Clone interaction: opposite growth | ASA HU IFN  Then I with CCyR.  Due to *JAK2* increase and BM features consistent with JAK2+ MPN  IFN+I+ASA  Good control of disease at 7 years from CML |
| Present report  Case 2 | 82/F | PMF, CML 7 yrs later | At the time of CML: WBC increase  Anemia  Splenomegaly  At the time of partial MR,  PTL increase (sign of JAK2+MPN) | At the time of CML:  Histology of CML+fibrosis  High cellularity myeloid hyperplasia  Reduced erythroid elements  Reduced MKs (mainly small-sized)  Grade 3 fibrosis  At the time of partial MR:  High cellularity myeloid hyperplasia clustering of variably-sized MKs  Grade 3 fibrosis  (features of JAK2+MPN with fibrosis) | t(9;22)  (q34;q11) present  *JAK2 V617F*+ 27% at the time of PMF;  at the time of CML, *BCR::ABL1*+ 95%  *JAK2 V617F*+ 30%.  At the time of partial MR  *BCR::ABL1* 18%  *JAK2 V617F* 15%  Clone interaction:  similar growth | HU for few weeks only; then I with partial MR; despite a good CML control, increase of PTL count, IFN added to I with DMR and reduction of *JAK2* 5%; good control of disease at 24 mo from CML diagnosis |

**Legends**: A: anagrelide; allo-SCT: allogenic stem cell transplant; AML: acute myeloid leukemia; AP: accelerated phase; ASA: acetylsalicylic acid; AWD: alive with disease; BM: bone marrow; BP: blastic phase; Bort: bortezomib; Bos: bosutinib; CyR: cytogenetic response; CCyR: complete cytogenetic response; CHR: complete hematological response; CML: chronic myeloid leukemia; CT: chemotherapy; Das: dasatinib; DIC: disseminated intravascular coagulopathy; DMR: deep molecular response; DOD: died of disease; ET: essential thrombocytemia; F: female; FU: follow-up; Fum: fumatinib; HCT: hematocrit; HU: hydroxyurea; Hydrox: Hydroxicarbamide; IFN: interferon; I: Imatinib; M: male; MKs: megakaryocytes; MM: multiple myeloma; MMR: major molecular response; Mo: month; MPN: myeloproliferative neoplasm; MR: molecular response; yrs: years; NA: not available; Nil; nilotinib; NP: not performed; NR: not reported; PB: peripheral blood; PET-MF: post-essential thrombocytemia myelofibrosis; PPV-MF: post-polycythemia vera myelofibrosis; Ph: Philadelphia; Phleb: phlebotomy; PMF: primary myelofibrosis; Pon: ponatinib; PTL: platelet; PV: polycythemia vera; RBC: red blood cell; RT: radiotherapy; Rux: ruxolitinib; Sun: sunitinib; Th: thalidomide; TKI: tyrosine kinase inhibitor; WBC: white blood cell;
